# Investigation of Sr-doped ZnO electron transport layers for perovskite solar cells: a combined DFT and SCAPS-1D study

**DOI:** 10.1039/d6ra05228b

**Published:** 2026-10-12

**Authors:** Shamima Ahmed, M. Mottakin, Aqib Adnan Shafin, Huda Abdullah, Abdulaziz K. Assaifan

**Affiliations:** a Department of Electrical and Computer Engineering, Boise State University 1910 University Drive Boise Idaho 83725 USA; b Institute of Advanced Research and Technology (IART), Dhaka Solar Ltd. Dhaka 1212 Bangladesh; c Department of Applied Chemistry and Chemical Engineering, Faculty of Engineering, Gopalganj Science and Technology University Gopalganj 8105 Bangladesh mottakin@gstu.edu.bd; d Department of Electrical, Electronic, and Systems Engineering, Faculty of Engineering and Built Environment, Universiti Kebangsaan Malaysia, UKM 43600 Bangi Selangor Malaysia; e Department of Biomedical Technology, College of Applied Medical Sciences, King Saud University P. O. Box 10219 Riyadh 11433 Saudi Arabia aassaifan@ksu.edu.sa

## Abstract

This study investigates strontium (Sr)-doped zinc oxide (ZnO) as an electron transport layer (ETL) for lead-free perovskite solar cells (PSCs) through a combined density functional theory (DFT) and SCAPS-1D simulation approach. The opto-electronics properties of pristine and Sr-doped ZnO were analysed using the mGGA functional implemented in CASTEP. The results revealed that Sr doping tunes the electronic structures of ZnO by reducing the band gap from 3.37 eV to 2.91 eV, while improving carrier mobility and optical absorption. Density of states (DOS) analysis confirmed that Sr doping enhances electron extraction capability. Optical investigations demonstrated high transparency in the visible region and tunable absorption behaviour suitable for photovoltaic applications. Device simulations were performed for a FTO/Sr–ZnO/FASnI_3_/spiro-OMeTAD/Au structure using SCAPS-1D. Optimization studies indicated that an ETL thickness of 150 nm and donor density of 1 × 10^20^ cm^−3^ provided the best performance, achieving an efficiency of 16.08% for the proposed PSCs. The study demonstrated that interface defect density, band alignment, series resistance, temperature, and back-contact work function strongly influence device performance. Overall, Sr-doped ZnO exhibits promising characteristics as an efficient ETL for stable and high-performance lead-free PSCs.

## Abstract 2

DFT calculation shows that Sr doping reduces the ZnO band gap, enhances carrier mobility and optical absorption. SCAPS-1D simulations demonstrate a 16.08% efficiency, highlighting Sr–ZnO as a promising ETL for perovskite solar cells.

## Graphical abstract

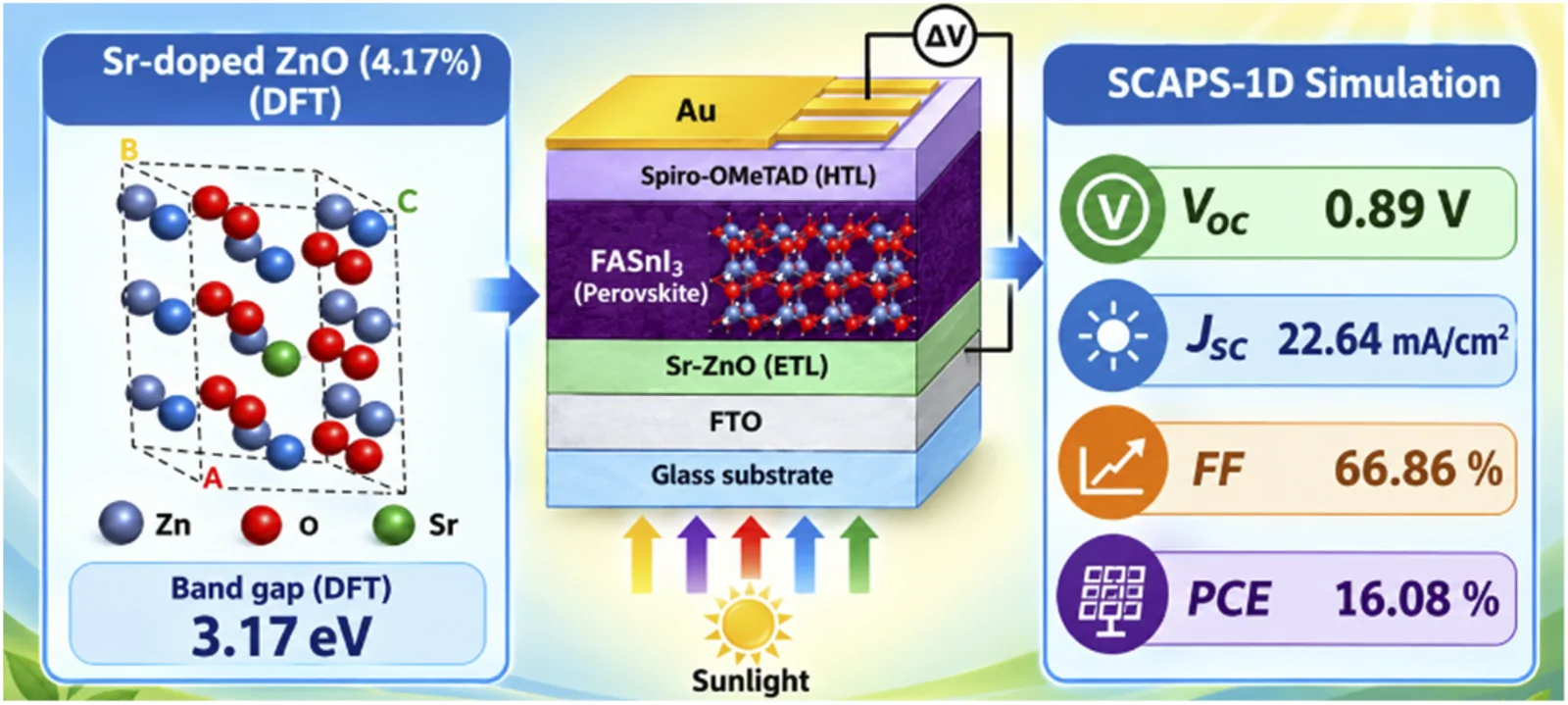

## 1 Introduction

The demand for sustainable and green energy technologies has been growing substantially to mitigate global warming. In this regard, the global energy consumption according to the International Energy Outlook has been projected to increase the amount of energy used by almost 50.8% by 2040.^1^ To address this growing demand, while at the same time reducing pollution in the ecosystem, renewable energy has become significantly important. However, using photovoltaic technology is attracting a lot of interest among different sustainable and renewable energy sources. The concept of solar energy was first developed in 1941 by Russel Ohl, who established and patented the first silicon-based solar cell. It showed improved performance compared to earlier selenium-based solar cells.^2^ From then on, advanced research has focused more on improving cost-effective silicon solar cells. However, the major evolution was the improvement of material quality that converted the polycrystalline silicon into monocrystalline silicon and effectively enhanced the overall device performance.^3^ This enabled silicon solar cells to achieve efficiencies of around 20%, making them commercially viable around the year 2000. Although they have been successful, the conventional silicon solar cells have some issues, such as high energy consumption during fabrication and limited potential for further efficiency improvements.^4^

Perovskite solar cells (PSCs) have become a promising photovoltaic solution over the past two decades to address the challenges of silicon solar cells. Recently, lead (Pb) based PSCs have made remarkable progress because of achieving a significant power conversion efficiency (PCE) of 26.1%.^5^ Additionally, they use up to 20% less material cost than traditional silicon-based solar cells.^6^ Despite their high performance, Pb-based PSCs are associated with significant toxicity concerns related to environmental safety and long-term stability. Lead is highly toxic, exposure to Pb can result in severe health issues, including kidney damage, hypertension, and reproductive disorders, as well as long-lasting environmental contamination.^7,8^ These concerns have driven the researchers to explore non-toxic substitutes to fabricate lead-free perovskite materials for photovoltaic implementations. Consequently, considerable investigations have been concentrated on Pb-free perovskite structures in which Pb is replaced by other metal ions. Several elements, including Sn,^9^ Ag,^10^ Bi,^11,12^ Sb,^13^ and Ti,^14^ have been investigated as potential substitutes. Amongst these candidates, Sn-based perovskites have shown notable potential optical and electronic characteristics due to their ionic radius and valence states are comparable to those of Pb. Pb-free PSCs still face several difficulties. Sn-based perovskites frequently experience rapid Sn oxidation due to unstable orbitals.^15^ FASnI_3_ was selected as the Pb-free perovskite absorber because the formamidinium (FA^+^) cation offers a suitable bandgap and relatively good thermal stability, making FA-based perovskites promising candidates for efficient photovoltaic applications.^16^ Till now, Pb-free PSCs exhibit very low efficiency. One effective approach to address these limitations is the proper selection of materials and application of doping, which can alter the electronic configuration and boost functional performance of the material.^17^ The efficiency of PSC is highly dependent on the electron transport layer (ETL) and the hole transport layer (HTL). These layers are important functions to enable the extraction and movement of charge carriers generated in the perovskite absorber.^18^ Therefore, it is important to select a suitable charge transport layer (CTL) and to improve device efficiency.^19^ Whereas, an ideal ETL should have a wide band gap to allow effective transmission of sunlight to the absorber layer, as well as exhibiting high electron mobility to facilitate accelerated charge transport.^20^ Moreover, optimal band energy level alignment between the ETL and the perovskite absorber is essential to reduce energy losses and enhance the overall effectiveness of the PSCs.^21^

Among the numerous ETL materials, ZnO shows a wide band gap (∼3.37 eV) and high exciton binding energy make it a promising material for optoelectronic devices.^22–24^ Despite these advantages, pure ZnO still has some limitations that restrict its performance. Rapid recombination of photogenerated charge carriers remains a major obstacle, diminishing charge transport performance. Additionally, ZnO responds to ultraviolet light and may suffer from photo corrosion under certain conditions.^25^ To overcome these drawbacks, researchers have explored the modification of ZnO through doping with various elements. Doping of ZnO can adjust its electrical and optical properties. For example, N, C, and S doping can enhance the quantum efficiency of ZnO-based photocatalytic systems.^26^ Metal dopants have also been widely investigated, including Ga,^27^ Al,^28^ and Fe. Theoretical analyses have demonstrated that Ag and Au doping can strengthen optical transitions in ZnO by modifying its electronic properties.^29^ Metal dopants can enhance device performance by extending charge-carrier lifetimes and minimizing electron recombination, allowing more carriers to transfer.^30^ Among the various dopants studied, alkaline earth metals have attracted attention as suitable candidates. Unlike transition metals, alkaline earth metals do not possess partially filled d-orbitals, which can help reduce the absorption threshold energy and improve optical properties.^31^ Sr has emerged as a promising dopant for ZnO, with its effects on thin-film wave filter devices being widely investigated.^32^ Sr dopant was selected because its incorporation into ZnO can narrow the band gap and modify the electronic structure, enabling effective tuning of the ETL's optoelectronic properties. Moreover, Sr^2+^ can substitute Zn^2+^ without charge mismatch, while its larger ionic size can induce favourable structural and electronic modifications. Vijayan *et al.* demonstrated that Sr-doped ZnO films synthesized *via* chemical bath deposition attained approximately 55% sensitivity to ethanol vapor at higher dopant concentrations.^33^ Prior research has demonstrated that Sr doping can enhance white-light emission in ZnO and create a red shift in the band gap.^34^ Further studies have investigated how Sr doping influences the optical characteristics of ZnO thin films fabricated by spray pyrolysis.^35,36^ Although several experimental studies have examined Sr-doped ZnO in different applications, theoretical investigations focusing on its role as an ETL in lead-free PSCs remain limited. In recent years, the PCE of tin-based PSCs using FASnI_3_ as the absorber has surpassed 10%.^37–41^ A key factor behind this improvement is the enhancement of the carrier diffusion length in the tin perovskite, improvement of the CTL, and device engineering. The novelty of the present work lies in the combined DFT and SCAPS-1D investigation of Sr-doped ZnO as an ETL for lead-free FASnI_3_-based PSCs, including the effect of Sr concentration on the electronic structure, effective mass, carrier transport, and overall device performance. Unlike previous reports that primarily focus on the synthesis and characterization of Sr-doped ZnO for other applications, this study establishes a direct relationship between Sr-concentration induced electronic-structure modification and photovoltaic device performance.

The device simulation offers a powerful strategy for understanding defect mechanisms and identifying pathways to improve photovoltaic performance. In this work, a hybrid computational approach is employed by integrating DFT calculations using CASTEP with device-level simulations performed using SCAPS-1D. The study begins with DFT-based analysis of Sr-doped ZnO to assess its structural, electronic, and optical properties. The proposed FTO/Sr–ZnO/FASnI_3_/spiro-OMeTAD/Au device is then analysed using SCAPS-1D. This approach helps determine the effectiveness of Sr-doped ZnO as ETL and supports the optimization of lead-free PSCs.

## 2 Computational methodology

### 2.1. DFT calculation

The optoelectronic properties were investigated using DFT calculations in CASTEP, employing the mGGA functional for exchange–correlation interactions. In this study, a hexagonal wurtzite ZnO structure, in which each Zn^2+^ ion is tetrahedrally coordinated with four neighbouring O^2−^ ions was created and geometrically optimised.^42^ A supercell configuration of 4 × 2 × 1 was constructed for the simulation, which contained a total of 24 zinc atoms (Fig. 1). The Brillouin zone sampling was performed using the Monkhorst–Pack scheme with a 1 × 1 × 2 *k*-point grid corresponding to the supercell structure. Self-consistent field (SCF) calculations were performed using a convergence threshold of 1 × 10^−5^ to achieve reliable electronic convergence. Geometry optimization was performed for the total simulation cell, permitting atomic positions as well as lattice parameters to relax until the convergence criteria were satisfied.^43^ The optimized structure obtained from this process was then used for further investigation of the electronic band structure, DOS, and optical properties of the material.

**Fig. 1:**
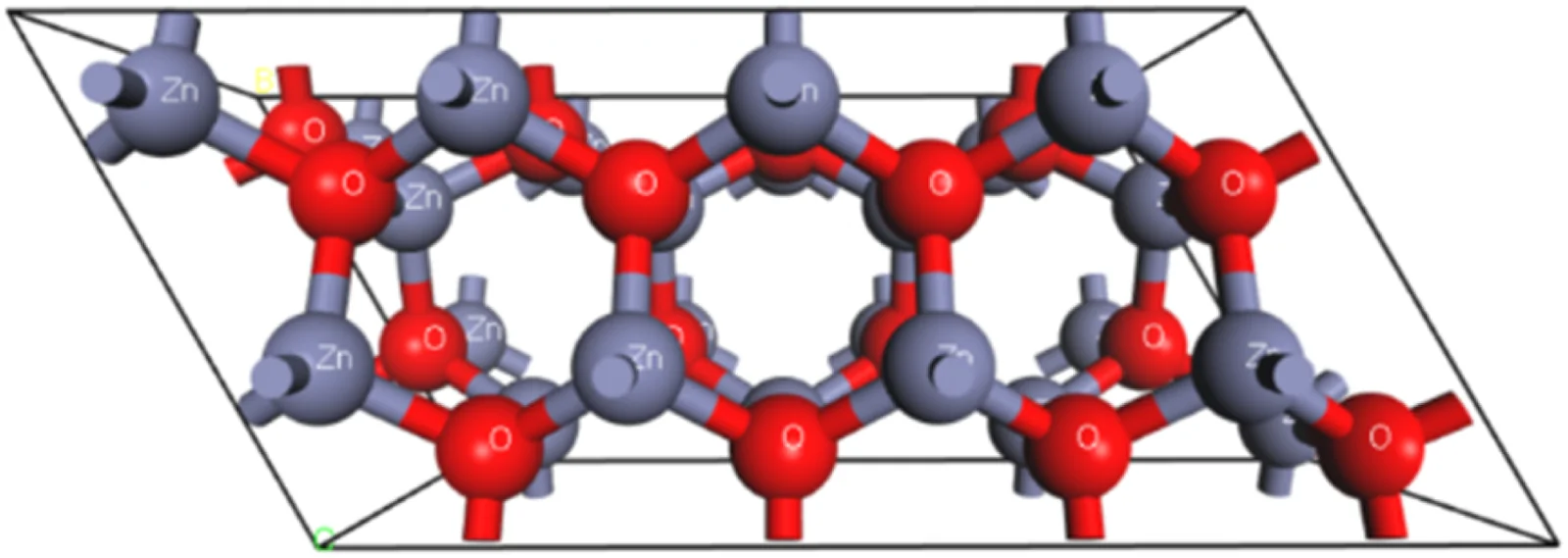
The optimized crystal structure of a 4 × 2 × 1 Sr–ZnO supercell. The grey and red spheres represent the zinc (Zn) and oxygen (O) atoms, respectively, illustrating the stable hexagonal wurtzite phase.

### 2.2. SCAPS-1D simulation

SCAPS-1D is widely used for thin-film solar cell modeling and optimization.^44,45^ SCAPS is now widely employed to investigate various solar cell technologies. It offers versatile simulation capabilities under both dark and illuminated conditions.^46^ SCAPS-1D was selected because it is well suited for simulating multilayer thin-film photovoltaic devices and enables efficient analysis of carrier generation, transport, and recombination within the device. It also allows in changing the key materials properties and device parameters to evaluate their influence on device performance. SCAPS can solve the basic semiconductor equation, namely Poisson's equation for electrons and holes (eqn (1)–(3)).^47^1
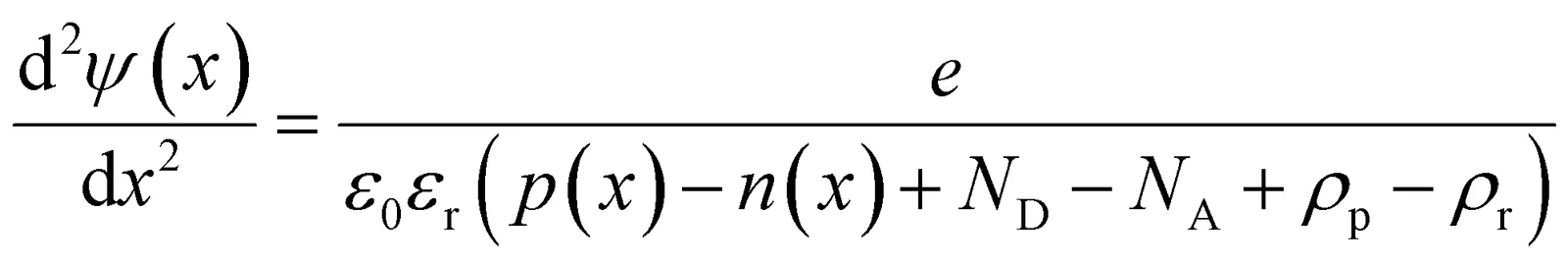
Here, *ψ* represents the electrostatic potential, *e* is the elementary charge, *ε*_r_ denotes the relative permittivity, and *ε*_0_ is the vacuum permittivity. *N*_D_ and *N*_A_ correspond to the concentrations of ionized donor and acceptor impurities, respectively, while *ρ*_n_ and *ρ*_p_ represent the electron and hole densities. The continuity equations for electrons and holes are:2
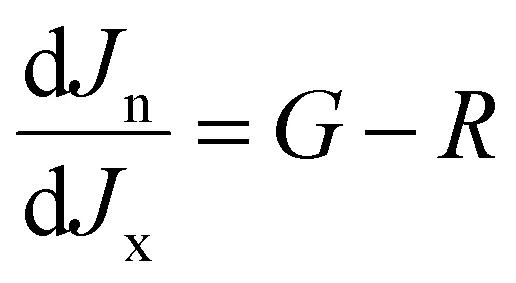
3
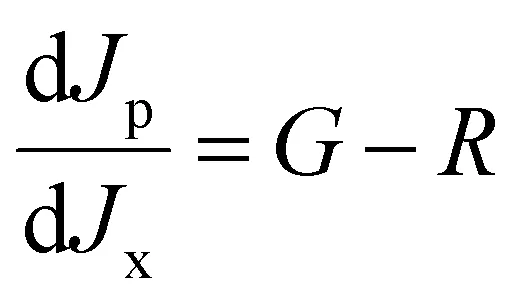


The transport of charge carriers in semiconductors is governed by drift and diffusion, as given in eqn (4) and (5):4
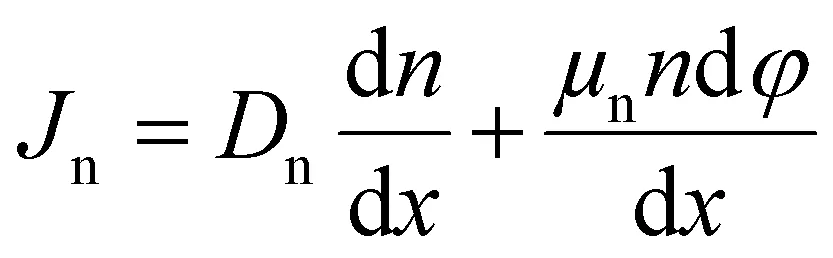
5
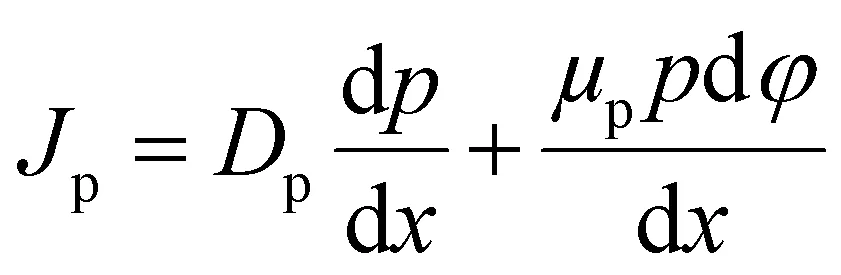
Here, *J*_p_ and *J*_n_ correspond to the current densities of holes and electrons, respectively, whereas *G* denotes the carrier generation rate. The simulation provides outputs such as *J*–*V* characteristics and quantum efficiency spectra, from which photovoltaic parameters, including *J*_SC_ and *V*_OC_ are obtained. The maximum power output is detected from the *J*–*V* data, and the fill factor (FF) is subsequently determined using the following equation.6
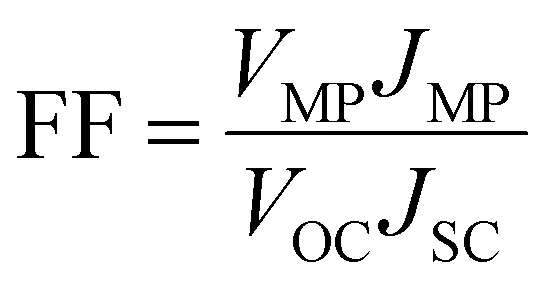


In this expression, *V*_MP_ and *J*_MP_ correspond to the voltage and current density at the maximum power point. The PCE is subsequently evaluated using the following relation:7
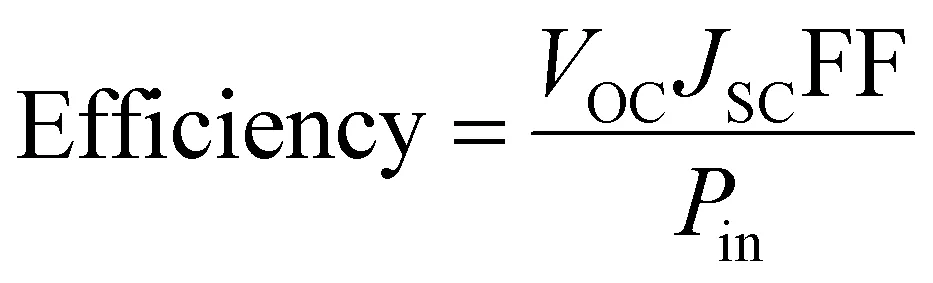


In this equation, *P*_in_ represents the incident light power supplied to the simulation. A detailed numerical investigation of the ZnO ETLs was conducted to evaluate their impact on solar cell performance and stability. Then the photovoltaic characteristics were calculated. In addition, the software allows the investigation of quantum efficiency (QE), spectral response, and carrier generation and recombination processes through both AC and DC electrical analyses. To evaluate the effectiveness of Sr-doped ZnO as an electron transport layer, a solar cell with the configuration FTO/Sr–ZnO/FASnI_3_/spiro-OMeTAD/Au was simulated. Simulations were carried out under AM 1.5G illumination with an incidence power density of 1000 W m^−2^ at 300 K.

### 2.3. Band gap correction using DFT + *U*

DFT has inherent limitations, including self-interaction errors and the absence of the derivative discontinuity in the exchange–correlation energy, which can cause charge delocalization and band gap underestimation, leading to inaccuracies in optical property predictions.^48^ The Hubbard correction (DFT + *U*) was implemented to improve consistency of band gap with experimentally observed values.^49^ However, the Hubbard *U* study was employed for the localized Zn-3d and O-2p orbitals using on-site coulomb correction parameters *U*_d_ and *U*_p_, respectively. The calculated band gap without Hubbard correction (*U* = 0) for pristine ZnO was 1.131 eV under both self-consistent field (SCF) convergence tolerances of 1 × 10^−5^ and 1 × 10^−6^, that delineates stable numerical performance and consistent convergence behaviour. The supercell parameters (4 × 2 × 1) and other DFT conditions were kept the same as those described in Section 2.1. When the Hubbard parameter *U*_d_ for Zn-3d states was implemented between the range, 1 eV to 10 eV, the band gap then demonstrated a gradual rise from 1.21 eV to 1.601 eV. This minor change indicates a weak contribution of Zn-3d orbitals to the valence band structure. As the *U*_p_ value increased from 1 to 6 eV, the band gap increased significantly from 1.601 eV to 3.374 eV. This substantial improvement originates from the dominant contribution of O-2p orbitals to the valence band maximum (VBM). The Hubbard correction shifts the O-2p states to the lower energy levels, thereby widening the band gap effectively (Fig. 2). The derived findings suggest that the electronic properties of ZnO are mainly controlled by O-2p states, whereas Zn-3d states play a comparatively subordinate role, influencing the band structure. Hence, the *U*_p_ parameter plays a vital role to adjust the band gap of Sr-doped ZnO accurately. On the other hand, *U*_d_ has a comparatively lower impact. For further doping studies, the selected computational parameters, including a plane-wave cutoff energy of 450 eV and the DFT + *U* values, were kept the same for 4.17% and 8.34% Sr doping.

**Fig. 2:**
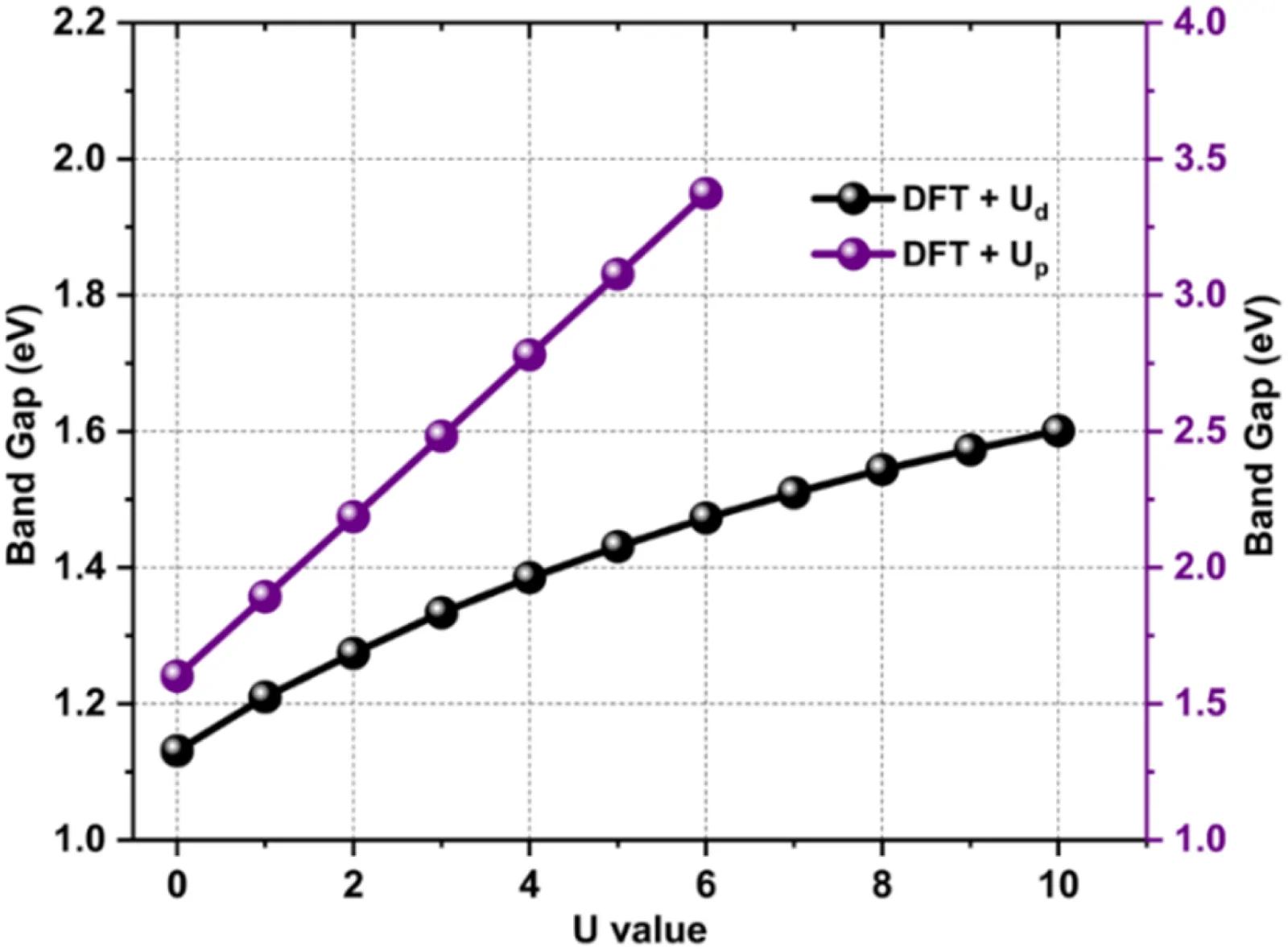
Influence of Hubbard *U* correction on the band gaps.

### 2.4. Band structure

The effect of Sr doping of ZnO was examined through band-structure calculations for both the pristine and doped systems. Fig. 3(a–c) depicts the electronic band structures. The energy gap was estimated from the distinction between the conduction band minimum (CBM) and valence band maximum (VBM). The calculated gap of 3.37 eV for undoped ZnO verifies its classification as a wide-band gap semiconducting material. It is suitable for optimal ETL in photovoltaic devices. Doping with Sr into the ZnO lattice causes a gradual reduction. For 4.17% Sr-doped ZnO, the band gap is reduced to 3.17 eV, whereas increasing the Sr concentration to 8.34% further lowers the band gap to 2.91 eV. The apparent drop in the band gap is mostly connected with alterations in the electronic structure caused by the incorporation of Sr into Zn lattice sites. Additionally, it generates additional energy states and modifies the electronic hybridization within the lattice. The VBM and CBM are located near the *Γ* (Gamma) point, suggesting a direct band gap nature of the material. Band dispersion near the edges facilitates electron extraction, while the reduced band gap enhances optical absorption and charge transport. Such tunability of the band structure through Sr doping makes ZnO a promising candidate for optimizing interface energy alignment in perovskite or thin-film photovoltaic devices. Charge carrier transport in semiconductor materials highly affects solar cell performance. The carrier mobility is closely related to the curvature of the electronic bands near the band edges, which determines the effective mass of electrons and holes. The effective mass m*is related to the curvature of the band structure and can be expressed as:8
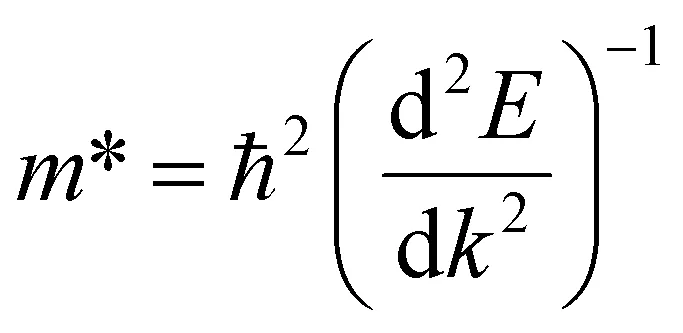
where ℏ is the reduced Planck constant^50^ and 
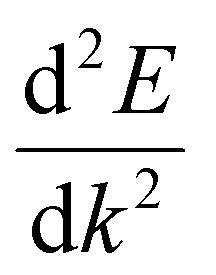
 represents the curvature of the energy band near the conduction or valence band edges.^51,52^ A higher band curvature corresponds to a lower effective mass and consequently higher carrier mobility.9
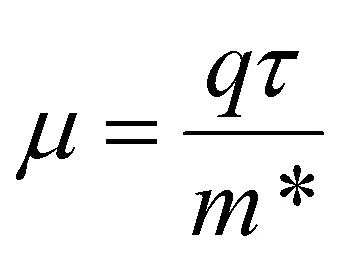


**Fig. 3:**
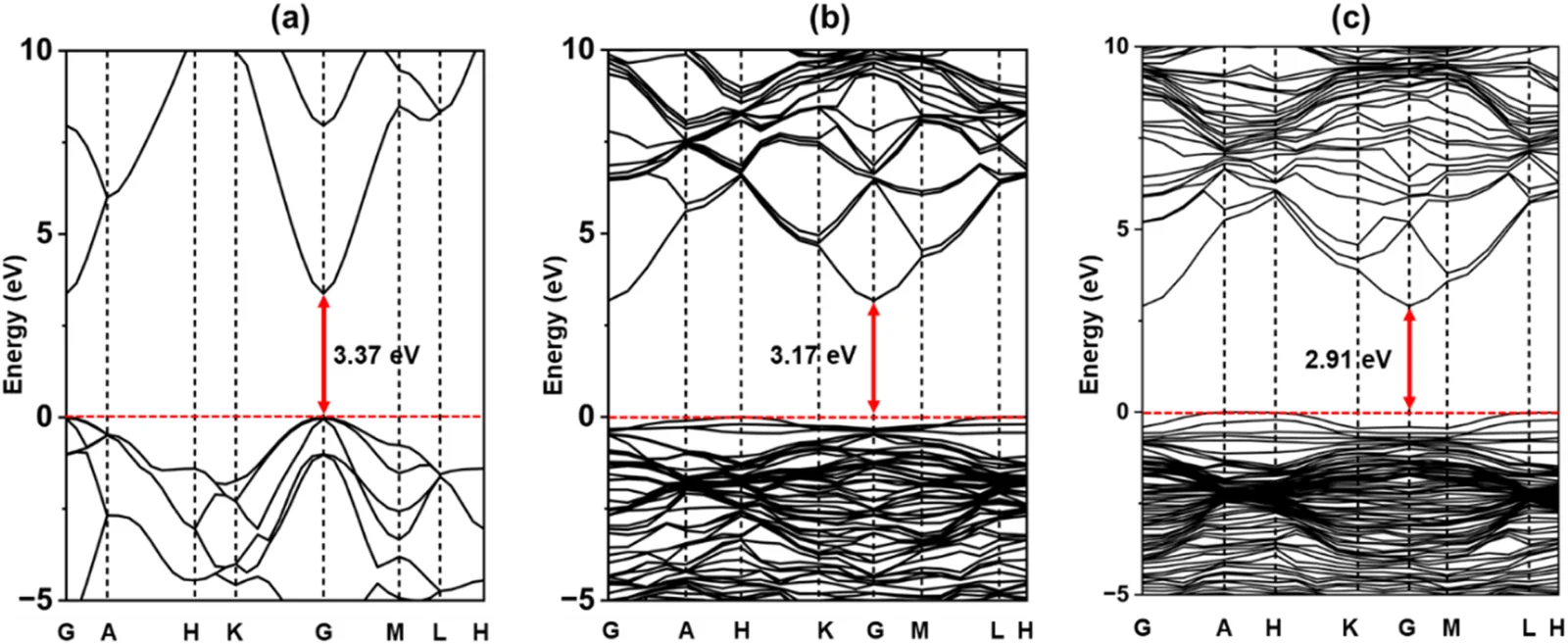
Band structure of ZnO polymorphs; (a) pristine ZnO, (b) 4.17% Sr doped ZnO, (c) 8.34% Sr doped ZnO.

Carrier mobility can be calculated using eqn (9), where *µ* is the mobility, *τ* is the scattering time, and *q* is the charge. The band dispersion observed near the *Γ* point suggests favourable carrier transport properties, which is advantageous for ETL applications. However, Fig. 3(a) illustrates the band structure of ZnO polymorphs. Fig. 3(b and c) shows the 4.17% Sr doped ZnO and 8.34% Sr doped ZnO, respectively. In the present case, doping increases the effective mass from 0.0364*m*_0_ for the pristine structure to 0.142*m*_0_ and 0.191*m*_0_ for the 4.17% and 8.34% Sr-doped structures, respectively, indicating flatter band dispersion near the *Γ* point. Consequently, the reduced band curvature leads to larger effective masses and contributes to the decrease in calculated mobility from 48.31 cm^2^ V^−1^ s^−1^ to 12.41 cm^2^ V^−1^ s^−1^ and 9.23 cm^2^ V^−1^ s^−1^, and hole mobility from 6.56 cm^2^ V^−1^ s^−1^ to 1.21 cm^2^ V^−1^ s^−1^ and 0.92 cm^2^ V^−1^ s^−1^ for pristine, 4.17% and 8.34% Sr-doped structures, respectively.

Thus, the lower mobility of the doped systems is a direct consequence of the modified band dispersion induced by doping. Despite the reduced carrier mobility, Sr doping was considered because it narrows the band gap and modifies the electronic structure, which can enhance the optical response and suitability of the material for photovoltaic applications. Thus, Sr doping represents a trade-off between carrier transport and helps in bandgap tuning, with the latter providing a potential benefit for overall device performance.

### 2.5. Density of states

To acquire more information about the electronic characteristics and orbital contributions of the constituent elements, the total and partial DOS were evaluated for both pure and Sr-incorporated ZnO, as presented in Fig. 4(a–c). The DOS analysis of undoped ZnO reveals that the upper VB region is predominantly derived from O-2p orbitals, whereas the lower CB region is mainly influenced by Zn-4s and Zn-3d states. The interaction and overlap between the electronic states of Zn and O play an important part in determining the electronic structure and for the relatively large band gap energy of ZnO. Upon introducing 4.17% Sr into the ZnO lattice, new electronic states emerge in the vicinity of the CB region. The PDOS results reveal a minor contribution from Sr-derived orbitals to the CB, leading to alterations in the electronic structure and a consequent decrease in the band gap energy. The incorporation of Sr modifies the distribution of electronic states through its interaction with the ZnO framework, thereby promoting improved charge transport characteristics.

**Fig. 4:**
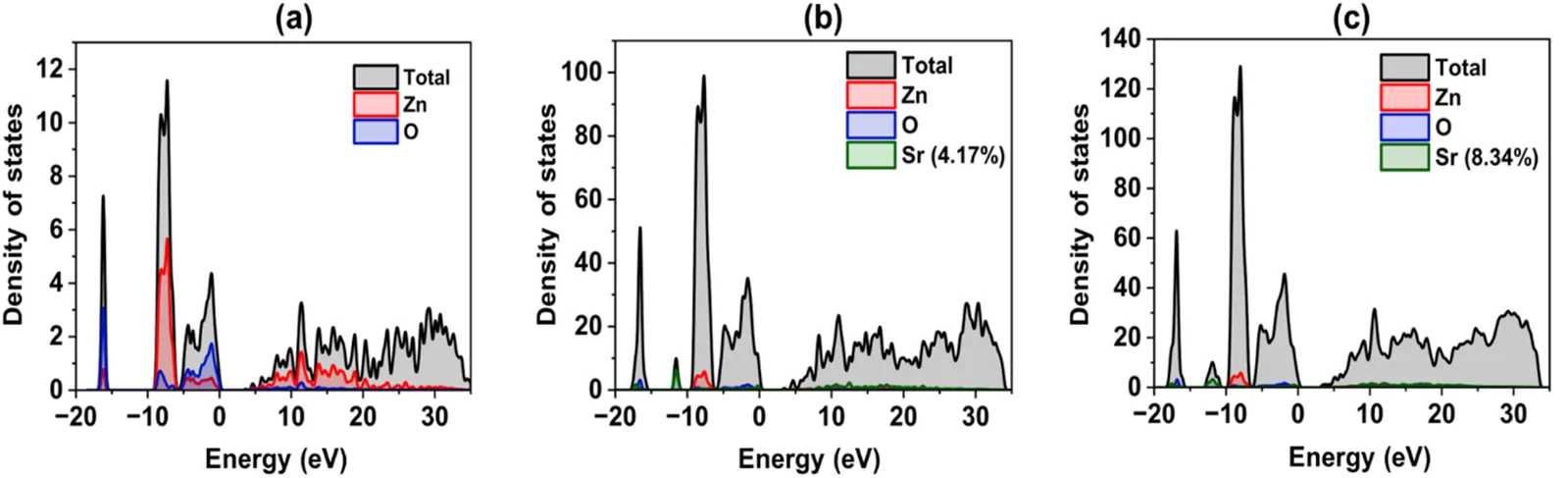
Density of states (DOS) and partial density of states (PDOS) of ZnO; (a) pristine, (b) 4.17% Sr doped, (c) 8.34% Sr doped ZnO.

At 8.34% Sr concentration, the increased density of states near both band edges indicates stronger dopant-host lattice coupling. At this doping level, the influence of Sr-related states on the conduction band (CB) is more significant, resulting in additional bandgap narrowing. The enhanced electronic-state density around the band edges provides more favourable pathways for carrier movement, which can contribute to improved electron mobility and enhanced electrical conductivity of the ETL. The DOS analysis reveals that Sr doping has a great impact on the ZnO structure by raising the density of accessible states near the CB. These changes contribute to the observed band gap reduction and improved electronic transport properties. Consequently, Sr-doped ZnO ETL can provide better band alignment and electron extraction capability.

### 2.6. Optical properties

To determine the applicability of ZnO in optoelectronic technologies, its optical response was analysed within the 300–900 nm wavelength range (corresponding to photon energies of 1.38–4.13 eV). This range is crucial for optoelectronic applications that ensure proper observation of the fundamental absorption edge^53^ and optical response of pristine and Sr doped ZnO structures. The reflectivity of the three structures is shown in Fig. 5(a). The reflectance spectrum exhibits relatively high values in the low-photon-energy region, followed by a gradual decline with increasing photon energy. Among the investigated samples, ZnO doped with 8.34% Sr displays the highest reflectivity. In comparison with undoped ZnO and the 4.17% Sr-doped structure, the 8.34% Sr-doped sample demonstrates greater optical reflection across the UV-visible spectral range. However, all structures exhibit similar decreasing trends at higher wavelengths, which suggests that the minimal reflection losses in the visible-NIR region. Fig. 5(b) illustrates the absorption spectra of the three samples pristine ZnO, 4.17% Sr doped ZnO and 8.34% of Sr doped ZnO, respectively. It is observed that the strong absorption is found in the UV region (300–400 nm), followed by a sharp decrease beyond 400 nm. Sr doping alters the electronic structure, resulting in a shift of the absorption edge. This investigation suggests that the ZnO, doped with Sr can be effectively tuned to get better light management in optoelectronic devices.

**Fig. 5:**
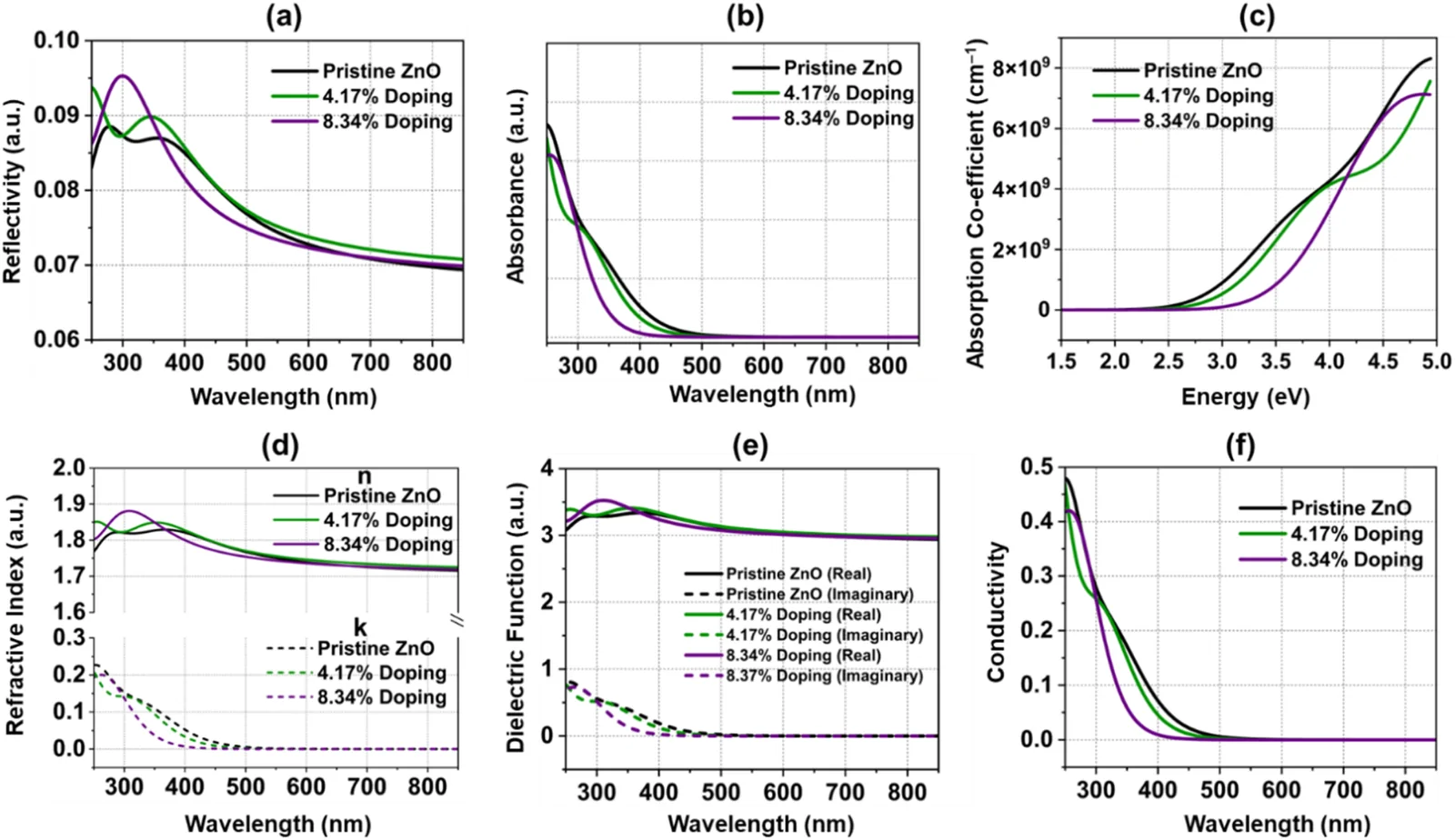
The optical properties of Sr–ZnO; (a) reflectivity, (b) absorption, (c) absorption coefficient, (d) dielectric function, (e) refractive index, (f) conductivity.

The absorption coefficient (*α*) was analysed using the following equation.^54^10
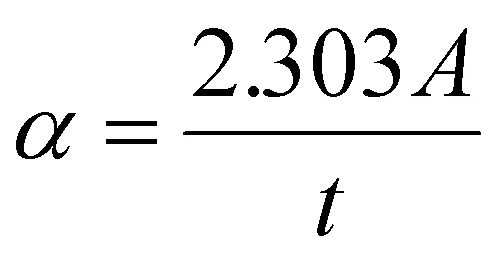
where *A* = absorbance, *t* = thickness of the film.^55^Fig. 5(c) demonstrates the absorption coefficient (*α*) as a function of photon energy. The absorption coefficient at visible range has reached the order of 10^7^. Among the structures, pristine ZnO exhibits a strong absorption edge in the UV region, while Sr-doped samples delineate gradual variation. This high absorption behaviour indicates that the ZnO can absorb light in the UV region. In the visible region, the absorption intensity decreases with increasing Sr doping concentration up to 500 nm wavelength. This trend may be associated with doping-induced modifications in the electronic structure and optical properties of ZnO. Beyond 500 nm, the absorption intensity becomes negligible. These optical characteristics suggest the potential suitability of Sr-doped ZnO as an ETL in n–i–p configured solar cells. Fig. 5(d) presents the refractive index (*n*) and extinction coefficient (*k*). Doping generally decreases the refractive index in the visible range (>400 nm). The 8.34% doping level shows the most significant deviation, showing a sharp peak value at low wavelengths. The extinction coefficient follows a decreasing trend and approaches zero in the visible region, confirming low optical loss and high transparency of the material.

The dielectric constants in Fig. 5(e) show strong UV responses, followed by a gradual reduction at longer wavelengths. However, the real part demonstrates the strong polarization at lower wavelengths. On the other hand, the imaginary part indicates optical absorption due to the variation of interband structure. It is observed that by doping Sr, material polarization and charge interaction can be modified because doping can change the dielectric behaviour. After 400 nm, the extinction coefficient (*k*) becomes almost zero which means the films are highly transparent in the visible range. Sr doped films have lower *k* values compared to pristine ZnO in the UV-visible region. The real part basically shows the material's ability to polarize, with a small change in peak intensity around 350 nm due to doping. In contrast, the imaginary part indicates energy loss, and its reduction with higher Sr doping suggests lower optical absorption in the near-UV region.

The real part mainly reflects polarization ability, with a small change in peak around 350 nm due to doping. The imaginary part shows energy loss, and its decrease with higher Sr doping means less optical loss in the near-UV region. The optical conductivity (*σ*) is shown in Fig. 5(f). A strong peak appears in the UV region due to intense interband electronic transitions, followed by a rapid decrease as the wavelength increases.^56^ At higher wavelengths, the doped samples exhibit slightly lower conductivity than pristine ZnO, indicating that Sr doping reduces the optical conductivity. This behaviour confirms that ZnO shows strong electronic transitions in the UV region while remaining stable in the visible range. This trend is consistent with the absorbance data, as increased transparency and a wider band gap led to fewer photon-induced electronic transitions within the measured range. Table 1 represents the reported optical and electronic properties for ZnO-based materials. It is seen that the properties varied significantly with dopant type and preparation method. Pristine ZnO generally exhibits a wide *E*_g_ of approximately 3.17–3.37 eV and electron mobility ranging from 5.2 to 50 cm^2^ V^−1^ s^−1^, with high optical transparency. Doping with Al, In, Si, Zr, and Sr modifies both the bandgap and carrier transport properties. The present mGGA calculations show that Sr incorporation reduces the ZnO bandgap, and the electron mobility also decreases. The corresponding absorption coefficient changes from high for pristine ZnO to moderate and lower values with increasing Sr concentration, demonstrating that Sr doping effectively tunes the optoelectronic properties of ZnO for photovoltaic applications.

**Table 1:** Summary of DFT-calculated structural, electronic bandgap, transport, and optical properties across undoped and donor-doped ZnO systems

No.	Material/dopant	Method & approach	Bandgap (eV)	Electron mobility (cm^2^ V^−1^ s^−1^)	Optical properties	References
1	Al–ZnO	RF sputtering	3.66	44.2	High optical transmission in visible wavelength range	57
2	ZnO	Spray pyrolysis	3.3	20–50	No noticeable visual photoluminescence observed	58
3	In–ZnO	Sol–gel spin coating	0.91	3.2–3.3	High optical transparency	59
4	Si–ZnO	Atomic layer deposition	3.17	5.2	High optical transparency (>80%)	60
6	ZnO	Atomic layer deposition	3.32	19.04	—	61
7	Zr–ZnO	Atomic layer deposition	3.43	19.0	High optical transparency (>90%)	62
8	ZnO	mGGA-rSCAN	3.37	48.31	High absorption coefficient	This work
9	4.17% Sr–ZnO	mGGA-rSCAN	3.17	12.41	Moderate absorption coefficient	This work
10	8.34% Sr–ZnO	mGGA-rSCAN	2.91	9.23	Lower absorption coefficient	This work

Overall, the results indicate that ZnO has high transparency in the visible-NIR region along with strong absorption in the UV region. The slight changes caused by doping suggest that the optical properties can be effectively tuned, making ZnO a suitable material for optoelectronic and photovoltaic applications.

### 2.7. Device simulation and calibration

To ensure the validity of any numerical simulation, it is necessary to verify that the simulated outputs closely represent experimentally observed device performance. Therefore, before analysing the proposed solar cell under different conditions, the simulation model was first calibrated using a previously reported experimentally fabricated PSC. In this work, the reference device structure was FTO/ETL/FASnI_3_/spiro-OMeTAD/Au, where Sr-doped ZnO was used as ETL. This calibration step was important to confirm that the SCAPS-1D simulation software could accurately reproduce the behaviour of the experimental device and provide reliable results for further optimization studies. For the practical device fabrication, the deposition conditions depend on the fabrication technique. Spray pyrolysis of the ZnO ETL generally requires an elevated substrate temperature (300–450 °C),^63^ whereas sputtering can be performed at relatively low temperatures (near room temperature to 200 °C).^64^ The FASnI_3_ absorber deposited at room temperature by spin coating requires subsequent thermal annealing for solvent removal and crystallization. Similarly, spiro-OMeTAD is deposited by solution processing,^65^ whereas the Au electrode is typically deposited by thermal evaporation or sputtering under vacuum. Thus, the deposition/annealing temperature is technique-dependent and should be optimized for each layer.

The complete device architecture and band alignment used for calibration is shown in Fig. 6(a and b). The front contact (FTO) acts as the transparent conducting oxide, allowing sunlight to enter the device, while the Au electrode collects the generated holes from the HTL side. The applied voltage (Δ*V*) across the contacts controls the separation and extraction of carriers. The Fig. 6(b) shows that the CB of the Sr–ZnO ETL aligns well with that of the FASnI_3_ absorber.

**Fig. 6:**
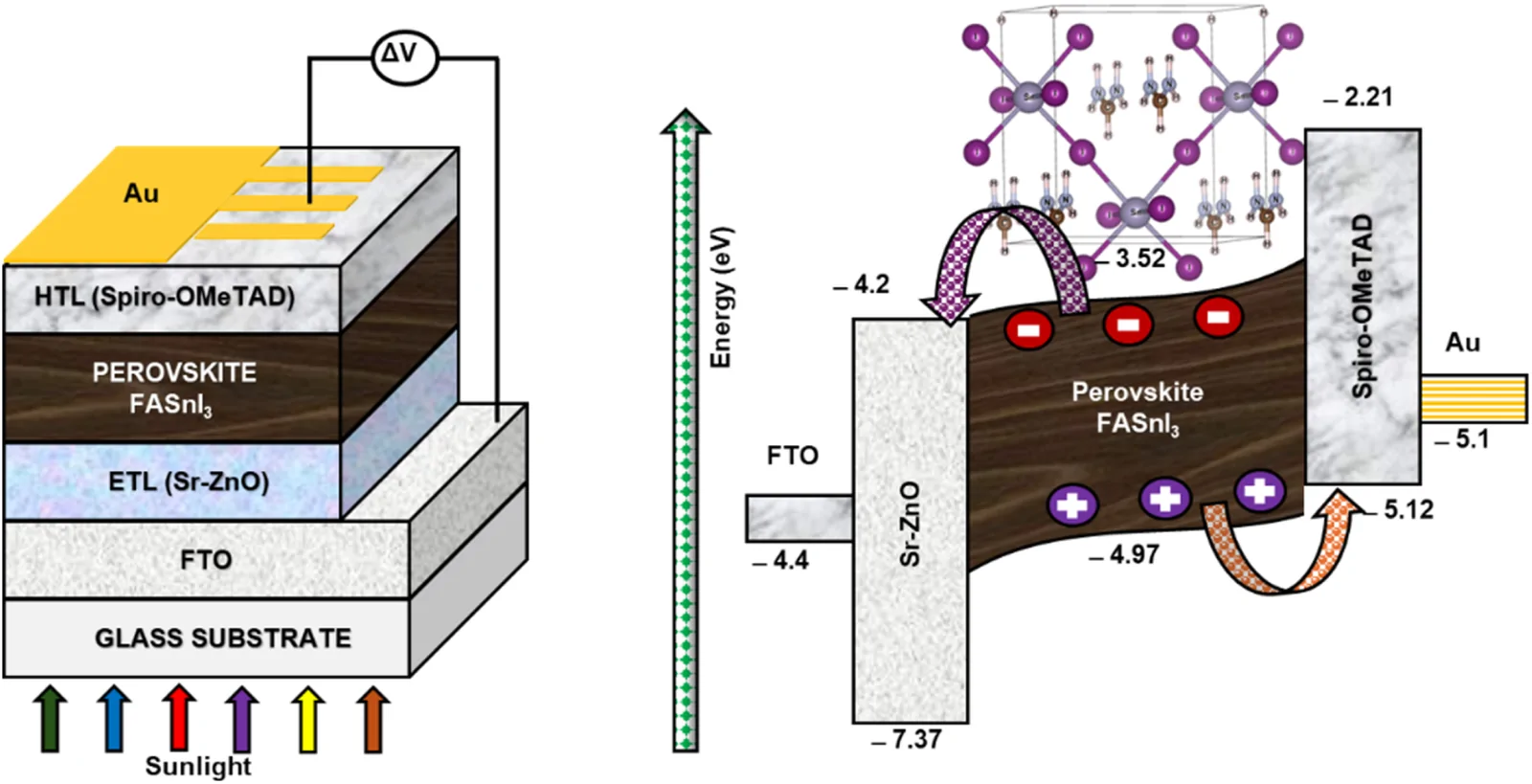
Schematic structure of the proposed PSC and energy band diagram.

Efficient energy-level matching at the interfaces promotes selective charge transport, suppresses carrier losses, and contributes to better photovoltaic performance. In this study, ETL was modelled using 4.17% Sr-doped ZnO, as this composition provides suitable band gap and stable transport characteristics. The photovoltaic performance of the device with 4.17% Sr doping was found to be very close to that of pristine ZnO, indicating that moderate Sr incorporation does not significantly alter the baseline operation of the device but improves the electronic properties required for stable simulation. For this reason, 4.17% of Sr-doped ZnO was selected as the reference ETL for calibration and all subsequent simulation analyses. The device performance was evaluated at 300 K with series resistance 1.65 Ω using the AM 1.5G spectrum with an irradiance of 100 mW cm^−2^, representing standard test conditions. The material parameters were used according to the values listed in Table 2. Some of these parameters were taken directly from the experimentally reported device, while the remaining values were adopted from previously published studies to complete the simulation model. For accurate calibration, several key parameters were adjusted carefully, including defect density, series resistance (*R*_s_), and shunt resistance (*R*_sh_), until the simulated current *J*–*V* characteristics closely matched the experimental results. These values were chosen to maintain proper carrier collection at both contacts and to reproduce the experimental device characteristics as closely as possible. A PSC, with FASnI_3_ was considered as a reference for this calibration.^66^ The comparison between experimental and simulated photovoltaic parameters is presented in Table 3.

**Table 2:** The material parameters were used in this work

Parameters	FTO^67^	Pristine and doped ZnO	FASnI_3_ (ref. 68)	Spiro-OMeTAD^69,70^
Thickness (nm)	200	50	500	100
*E*_g_ (eV)	3.2	DFT	1.45	2.91
*χ* (eV)	4.4	4.2	4.1	2.2
*ε*_r_	9	DFT	8.2	3.0
*N*_C_ (cm^−3^)	2.2 × 10^18^	3.70 × 10^18^	1 × 10^18^	2.8 × 10^19^
*N*_V_ (cm^−3^)	1.80 × 10^19^	1.80 × 10^19^	1 × 10^18^	1 × 10^19^
*µ*_n_ (cm^2^ V^−1^ s^−1^)	90	DFT	22	0.00012
*µ*_h_ (cm^2^ V^−1^ s^−1^)	90	DFT	22	0.0002
*N*_A_ (cm^−3^)	0	0	1.0 × 10^16^	1.0 × 10^18^
*N*_D_ (cm^−3^)	7 × 10^20^	1.0 × 10^18^	1.0 × 10^16^	0
*N*_t_ (cm^−3^)	1.0 × 10^14^	1.0 × 10^14^	1.0 × 10^16^	1.0 × 10^14^

**Table 3:** Comparison between the cell performance parameters of experimental and simulated PSCs

PSC	*J*_SC_ (mA cm^−2^)	*V*_OC_ (V)	FF (%)	PCE (%)
Experimental	22.87	0.84	70.9	13.61
Simulated	22.65	0.87	58.79	13.74

Although the simulated and experimental values of *J*_SC_, *V*_OC_, and PCE are in close agreement, a noticeable discrepancy is observed in the FF. The experimental and simulated values of FF are 70.9% and 58.79%, respectively. In this work, SCAPS-1D calibration primarily focused on reproducing the *J*_SC_, *V*_OC_, and overall PCE. Adjusting the series and shunt resistances to achieve a closer match in FF substantially altered *J*_SC_, *V*_OC_, and the overall PCE. Therefore, physically consistent device parameters were retained rather than forcing the simulated FF to match the experimental value. The simulated *V*_OC_ (0.87 V), *J*_SC_ (22.65 mA cm^−2^), and PCE (13.74%) show close agreement with the corresponding experimental values of 0.84 V, 22.87 mA cm^−2^, and 13.61%, respectively. The lower simulated FF can be attributed to the sensitivity of the maximum-power-point characteristics to resistive losses, recombination processes, and interface-related parameters, together with the limitations of the idealized one-dimensional SCAPS-1D model in capturing fabrication-dependent effects present in experimentally fabricated devices. The minimal PCE variation confirms the reliability of the SCAPS-1D model in replicating the reference device performance. Fig. 7 compares the experimental and simulated *J*–*V* curves. This approach validates the simulation approach adopted in this work. Therefore, the calibrated model can be considered reliable for investigating the impact of Sr–ZnO ETL properties and for further optimization of the FASnI_3_-based PSCs.

**Fig. 7:**
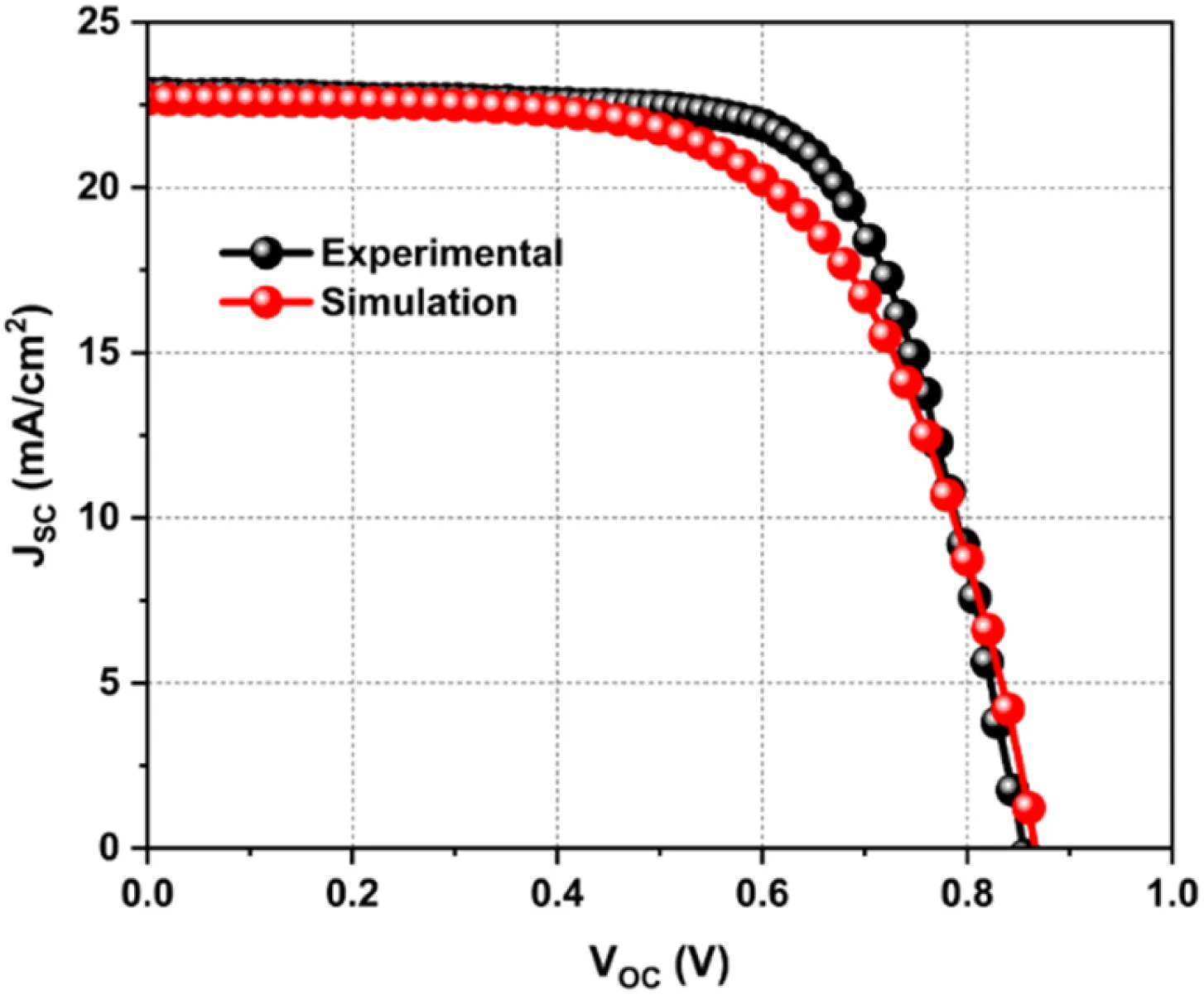
Comparison between the *J*–*V* curves of experimental and simulated PSCs.

### 2.8. Effect of thickness

Layer thickness significantly influences PSC performance by affecting charge generation and collection. Therefore, optimizing the thickness of the Sr-doped ZnO ETL is essential. The electron concentration in Sr-doped ZnO strongly affects PSC efficiency through its impact on carrier transport, resistive losses, and recombination processes. Fig. 8(a–d) presents the photovoltaic parameters with varying ETL thickness and doping level. In this study, a broad donor density range of 10^12^ to 10^20^ cm^−3^ was systematically investigated, which is commonly adopted in SCAPS-1D simulations for device optimization process. Importantly, these values are also consistent with experimentally reported carrier concentrations in doped ZnO-based films. Shamima *et al.* studied on Ga-doped ZnO sputtered thin films and reported a carrier concentration of 1.586 × 10^19^ cm^−3^.^71^ In addition, Doppler Broadening Spectroscopy (DBS) studies of Al-doped ZnO (AZO) films have reported defect concentrations of 10^18^ to 10^19^ cm^−3^ for epitaxial and polycrystalline films and up to 10^20^ cm^−3^ for partially amorphous films.^72^ These experimental observations support the physical relevance of the donor and defect-density range considered in this simulation.

**Fig. 8:**
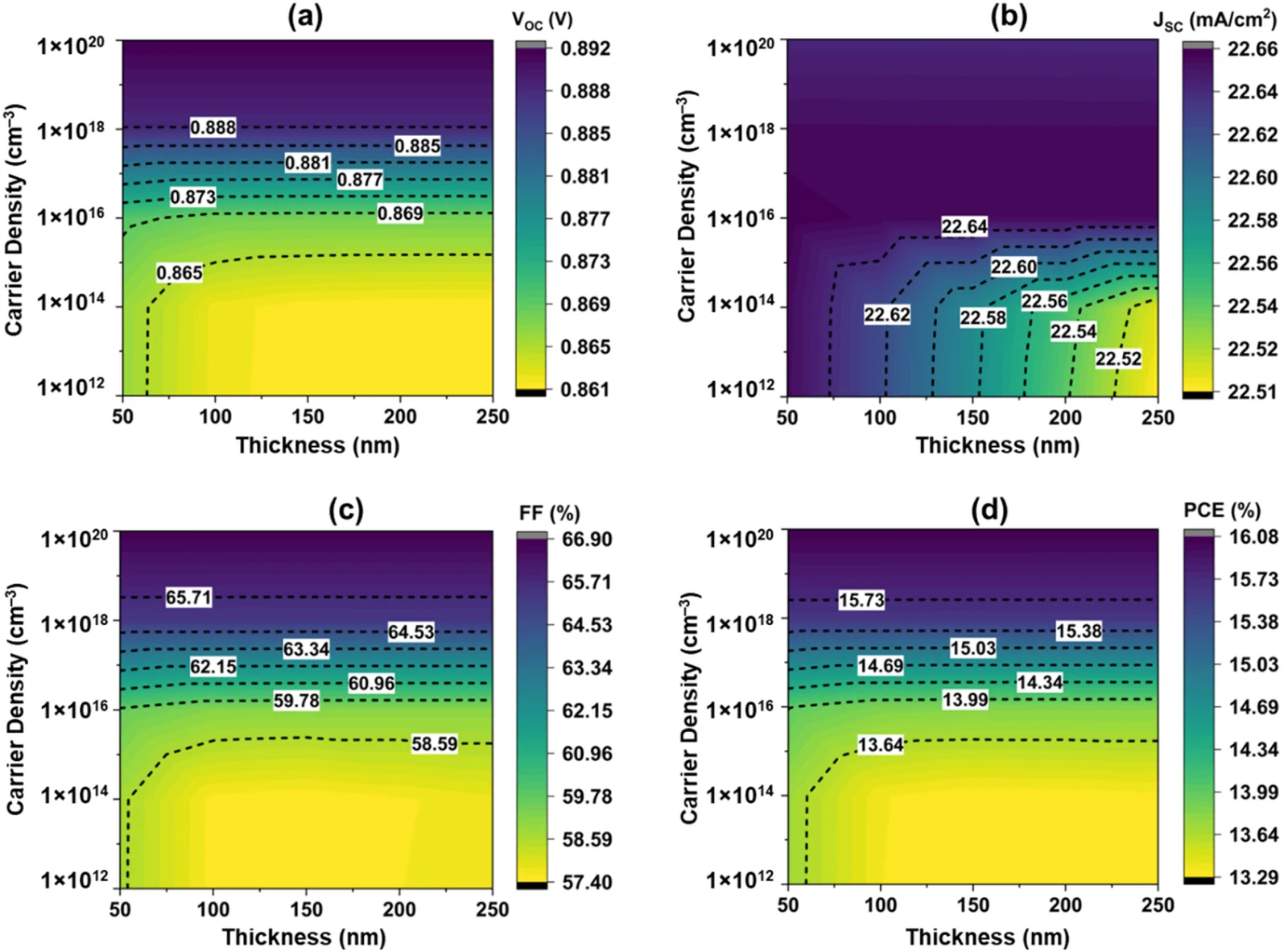
(a)–(d) Variation in the solar cell properties with the thickness and carrier density of Sr–ZnO.

At an ETL thickness of 50 nm, increasing the donor concentration from 1 × 10^12^ to 1 × 10^20^ cm^−3^ causes only minor changes in *V*_OC_ and *J*_SC_, which remain close to 0.87–0.89 V and 22.63–22.65 mA cm^−2^, respectively. However, the fill factor rises markedly from 58.68% to 66.87%, resulting in an increase in PCE from 13.71% to 16.08%. The enhanced performance is primarily associated with improved conductivity and lower series resistance, enabling more effective carrier extraction and transport. On the other hand, when the donor density is maintained at 1 × 10^12^ cm^−3^ and the ETL thickness increases from 50 nm to 250 nm, a slight reduction in both *V*_OC_ and *J*_SC_ is observed. Meanwhile, the FF decreases from 58.68% to 57.79%, resulting in a decline in PCE from 13.71% to 13.34%. The degradation in device performance with increasing thickness is primarily associated with the increased transport path for charge carriers, which elevates the series resistance and limits carrier extraction efficiency. An optimal trade-off between conductivity and transport losses is achieved at an ETL thickness of 150 nm combined with a donor density of 1 × 10^20^ cm^−3^ yielding a maximum PCE of 16.08%. Furthermore, the contour plots reveal that within the thickness range of approximately 120–180 nm, the PCE remains nearly constant (∼16.07–16.08%), indicating a stable performance window. This suggests that moderate variations in thickness within this range do not significantly affect device efficiency. Higher donor concentrations enhance carrier extraction and suppress recombination, whereas overly thick ETLs increase transport losses. An ETL thickness of 150 nm and donor density of 1 × 10^20^ cm^−3^ provide the best balance, yielding optimal device performance.

### 2.9. Effect of bulk and interface defect (Sr–ZnO/FASnI_3_)

Defect density at the bulk and the Sr–ZnO/FASnI_3_ interface plays an important function in determining the overall performance of the PSC, as these defects introduce recombination centres that facilitate Shockley–Read–Hall (SRH) processes and thereby limit carrier transport.^73–75^ As illustrated in Fig. 9, a systematic increase in defect density leads to a noticeable deterioration in photovoltaic parameters, with interface defects showing a much stronger influence compared to bulk defects in the absorber layer. In particular, when the interface defect density at the Sr–ZnO/FASnI_3_ junction is increased from 10^12^ to 10^20^ cm^−2^, the device performance degrades significantly. Increased defect density results in significant carrier reductions because of greater interface recombination, which in turn drastically reduces *J*_SC_. The *V*_OC_ reduction is mainly attributed to increased nonradiative recombination arising from interface trap states, which elevate the recombination current and weaken the built-in potential.^76,77^ Quantitatively, *V*_OC_ drops from 0.87 V to 0.83 V and *J*_SC_ decreases from 22.65 to 19.51 mA cm^−2^ under high defect conditions. This recombination loss also leads to a severe reduction in fill factor from 58.79% to 42.72% and a corresponding decline in power conversion efficiency from 13.74% to 8.24%, indicating strong suppression of charge extraction efficiency. The observed behaviour is associated with intensified trap-assisted recombination originating from lattice mismatch and interfacial chemical disorder at the Sr–ZnO/FASnI_3_ boundary, which shortens carrier lifetime and limits effective carrier collection.^70^ Although bulk defect variations also influence device characteristics, their impact remains comparatively less pronounced, confirming that interface quality dominates the recombination dynamics in this system. It has been reported that pristine ZnO contains intrinsic defects, particularly oxygen vacancies and zinc interstitials, which can promote interfacial recombination and perovskite degradation.^78,79^ Doping ZnO can mitigate these defects and tune its electronic structure, thereby improving charge extraction and overall device performance.^80^ Overall, the simulation results clearly highlight that precise control and passivation of interface defect states at the Sr–ZnO/FASnI_3_ junction are essential to reduce recombination losses and achieve high efficiency.

**Fig. 9:**
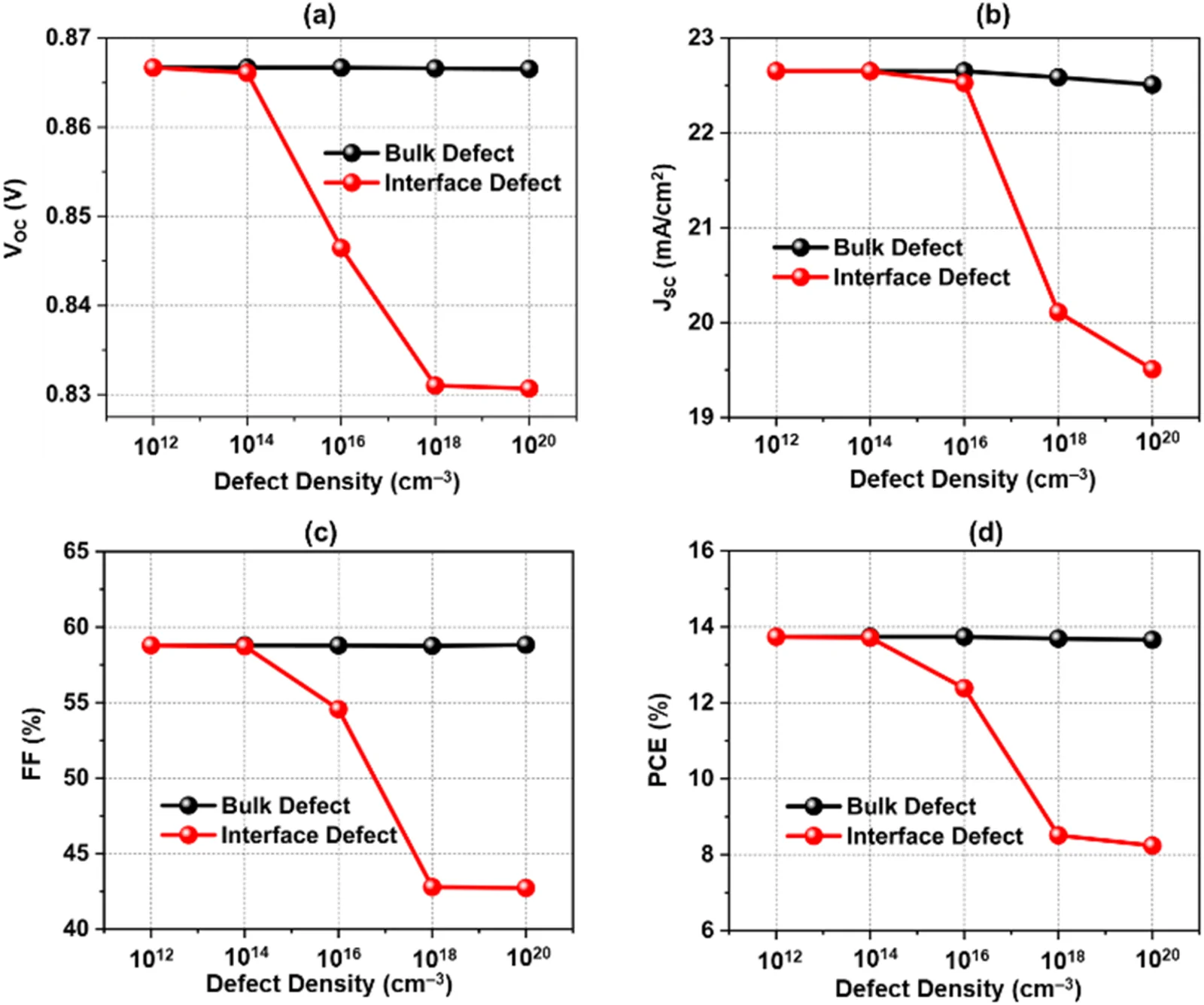
(a)–(d) Change in the solar cell parameters at the Sr–ZnO/FASnI_3_ junctions.

### 2.10. Generation and recombination rate

Fig. 10 depicts the variation of carrier generation and recombination behaviour interface defect densities ranged from 10^12^ to 10^20^ cm^−2^. It is observed from Fig. 10(a) that the generation rate remains almost constant for all considered defect densities. The photogenerated charge carriers are mainly generated in absorber layer, where light absorption is most effective because of its appropriate band gap and strong ability to absorb light.^81^ However, most of the generation is observed near to the absorber/ETL junction. In addition, it is consistent with the optical field distribution inside the device. Conversely, ETL shows very little or almost negligible carrier generation due to its wide band gap ability that permits the majority of incoming photons to pass through without absorption. This phenomenon ensures that instead of depending on the interface defect density, the generation process is mainly controlled by the intrinsic optical properties of the absorber materials.

**Fig. 10:**
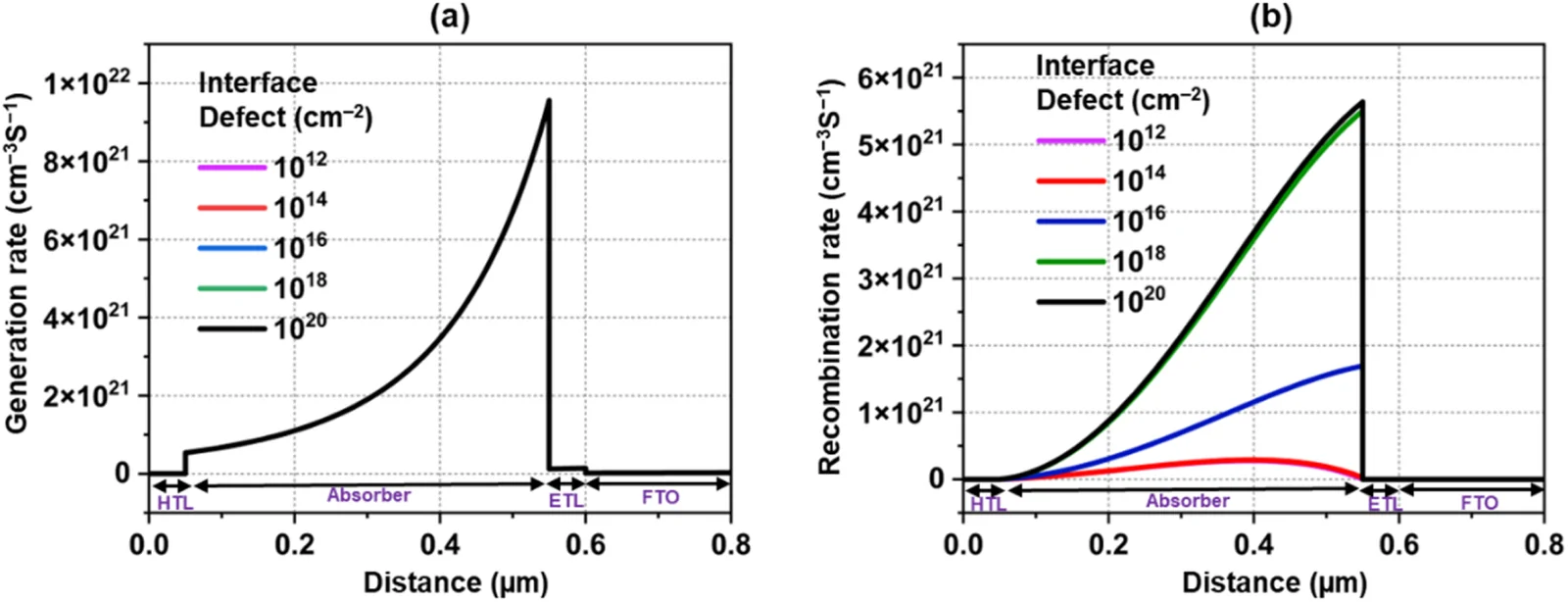
Variation of device parameters for (a) carrier generation and (b) recombination.

On the other hand, Fig. 10(b) highlights that recombination strongly depends on the interface defect density. At lower defect densities from 10^12^ to 10^20^ cm^−2^, the recombination rate remains very low throughout the device that clearly demonstrates the efficient carrier transport and minimal loss through defect-assisted processes. In such cases, most of the photogenerated carriers can move toward their respective transport layers without significant recombination. However, as the interface defect density increases to 10^18^ and 10^20^ cm^−2^, a remarkable increase in recombination is observed, notably concentrated near the absorber/ETL interface region. This increase is mainly due to the formation of many defect states at the interface, which act as trap centres for both electrons and holes. These trap states enhance Shockley–Read–Hall (SRH) non-radiative recombination, leading to a higher probability of carrier capture and annihilation before collection. As a result, a significant portion of photogenerated carriers is lost, which reduces carrier extraction efficiency, decreases photocurrent contribution, and ultimately causes considerable reduction in overall device performance.

### 2.11. Effect of band offset at ETL/absorber

The band alignment at the ETL/absorber interface serves an important function in governing charge extraction and overall photovoltaic performance in PSCs. In particular, the conduction band offset (CBO) significantly influences electron transport dynamics and interfacial recombination losses. The CBO is defined as:

11 Δ*E*_c_ = *χ*_absorber_ − *χ*_ETL_

where *χ*_absorber_ and *χ*_ETL_ represent the electron affinities of the absorber and ETL layers, respectively. Numerical modelling and experimental validation indicate that the sign and amount of this offset produce either a cliff, a flat band, or a spike, directly regulating the *V*_OC_, *J*_SC_, and FF.^82,83^ In this study, the absorber electron affinity was fixed at 4.1 eV, while the ETL electron affinity was systematically varied to obtain CBO values ranging from +0.30 eV to −0.30 eV. This tuning allows a detailed investigation of its effect on interfacial band alignment and device performance. Fig. 10 illustrates the CB alignment at the ETL/absorber interface for different CBO values. As the CBO changes from positive to negative, the interface transitions from a spike-type alignment to a cliff-type alignment due to the relative shift in the ETL CB position. For positive CBO values (+0.10 to +0.30 eV), the ETL CB lies above that of the absorber, forming an energy barrier (spike) at the interface. This occurs because the ETL has a lower electron affinity than the absorber, causing electrons to experience a potential barrier during transfer. A moderate spike (+0.10 eV) is found to be beneficial, as it suppresses back electron flow from the ETL to the absorber and reduces interfacial recombination, thereby enhancing device performance. However, when the spike becomes too large (+0.20 to +0.30 eV), it hinders electron extraction, leading to carrier accumulation at the interface and increased series resistance, which ultimately reduces the fill factor. At zero CBO (0 eV), nearly perfect band alignment is achieved between the ETL and absorber layers, resulting in a flat-band configuration. In this case, electrons can be transported without encountering any energy barrier. However, the absence of a selective barrier slightly increases interfacial recombination compared to the optimal small positive offset, leading to marginally lower device performance.^84^

For negative CBO values (−0.10 to −0.30 eV), the ETL CB shifts below that of the absorber, forming a cliff-like alignment. This situation arises when the ETL electron affinity is higher than that of the absorber.^85^ Although electron extraction becomes energetically favourable, the absence of a blocking barrier enhances interfacial electron accumulation, which increases the probability of electron–hole recombination at the interface. As the negative offset increases in magnitude, the cliff becomes deeper, further intensifying recombination losses and degrading key photovoltaic parameters such as open-circuit voltage and fill factor.^86^ The inset of Fig. 11 clearly highlights the evolution of the interface from spike to cliff configuration, emphasizing the strong dependence of band alignment on ETL electron affinity. Overall, the results indicate that a slight positive CBO (∼+0.10 eV) provides the most favourable balance between efficient electron extraction and suppression of interfacial recombination. In contrast, both large positive spikes and negative cliffs lead to unfavourable charge transport conditions and reduced device efficiency.^86^

**Fig. 11:**
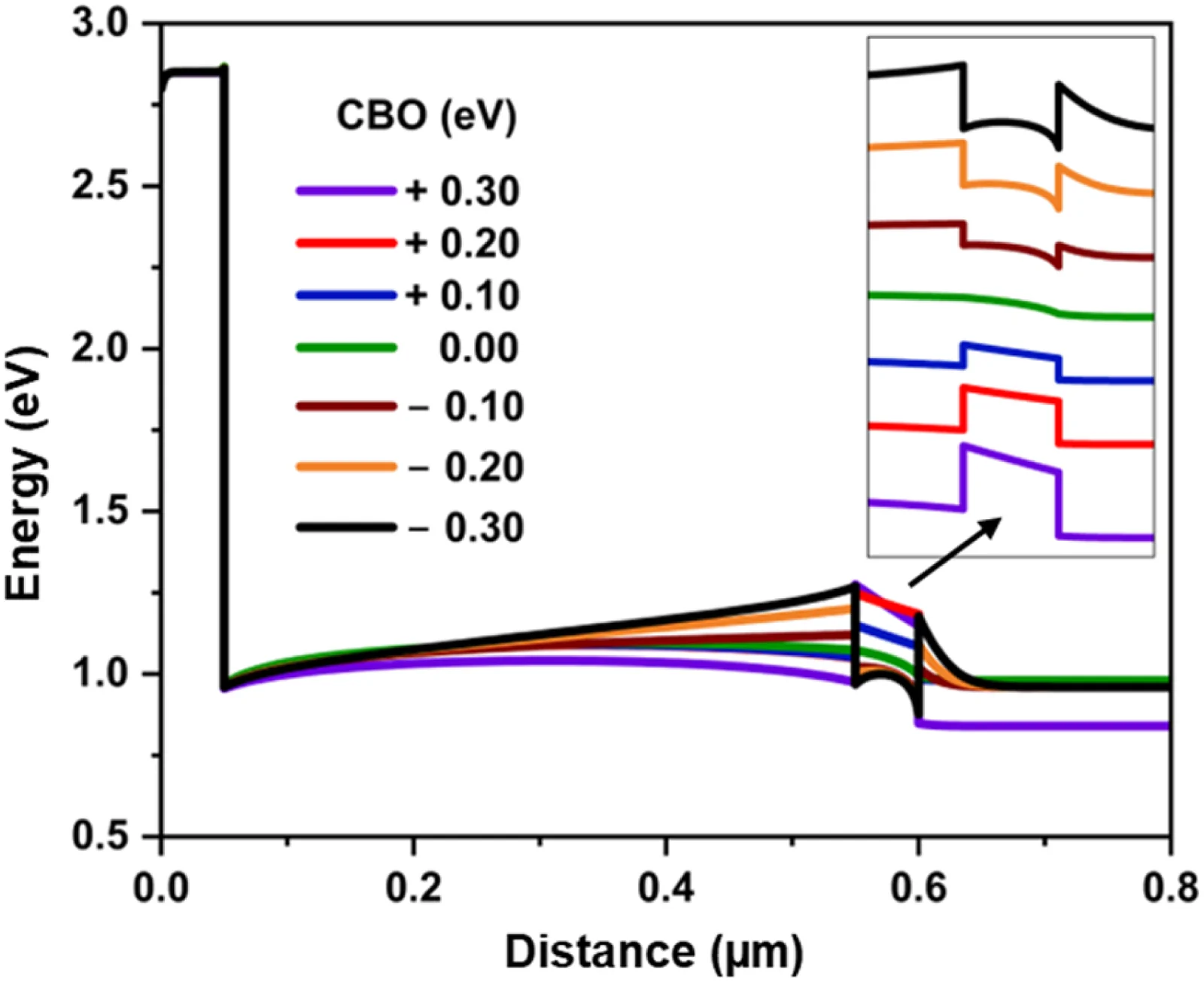
Variation of conduction band offset at ETL/absorber interface.

These findings are consistent with previous reports, which demonstrate that a small positive spike at the ETL/perovskite interface optimizes charge selectivity and minimizes recombination losses, whereas cliff-like offsets significantly deteriorate photovoltaic performance due to enhanced interfacial recombination.

### 2.12. Effect of series (*R*_series_) and shunt resistance (*R*_shunt_)

Series and shunt resistances critically affect solar-cell efficiency by controlling *J*–*V* behaviour and internal power losses.^87^ In general, efficient solar cells require low *R*_series_ and high *R*_shunt_ values to ensure effective charge transport and minimum power loss. Shunt resistance represents unintended parallel current pathways within the device. Low *R*_shunt_ promotes leakage current and reduces the FF and *V*_OC_, whereas a high *R*_shunt_ minimizes leakage and improves overall device performance. However, in real devices, the *R*_shunt_ value is often lower than the ideal case due to unavoidable leakage currents and morphological imperfections in the functional layers.^88^ These imperfections can facilitate unwanted current flow and reduce the overall device performance. Series resistance originates through the semiconductor layers, interfacial contacts, and electrodes. As a result, an increase in *R*_series_ adversely affects the photovoltaic parameters and reduces the overall PCE. Since *R*_series_ are highly dependent on the quality of the electrical contacts between the CTL and the metal electrode, evaluating its effect is essential for understanding realistic device behaviour.

To investigate these effects, the *R*_series_ was varied from 1 Ω cm^2^ to 5 Ω cm^2^, while the *R*_shunt_ was adjusted from 1000 Ω cm^2^ to 5000 Ω cm^2^. The simulation results reveal that even a slight increase in *R*_series_ significantly deteriorates the device performance. In particular, the FF decreased markedly from 60.6% to 53.5% for the PSC employing Sr–ZnO as the ETL. In contrast, the *V*_OC_ remained nearly unchanged, whereas the *J*_SC_ showed only a slight reduction from 22.65 mA cm^−2^ to 22.62 mA cm^−2^. This decline results from energy losses, causing a reduction in *J*_SC_. Consequently, the overall PCE decreased noticeably from 14.17% to 12.5% with increasing *R*_series_.

On the other hand, increasing the *R*_shunt_ improved the device performance by suppressing leakage current pathways within the device. As the *R*_shunt_ increased, the FF improved from 57.3% to 58.5%, while the PCE increased from 13.3% to 13.6%. Meanwhile, both *V*_OC_ and *J*_SC_ exhibited only negligible changes with increasing *R*_shunt_ resistance. Fig. 12 shows variation of device parameters for (a and b) series and (c and d) shunt resistance.

**Fig. 12:**
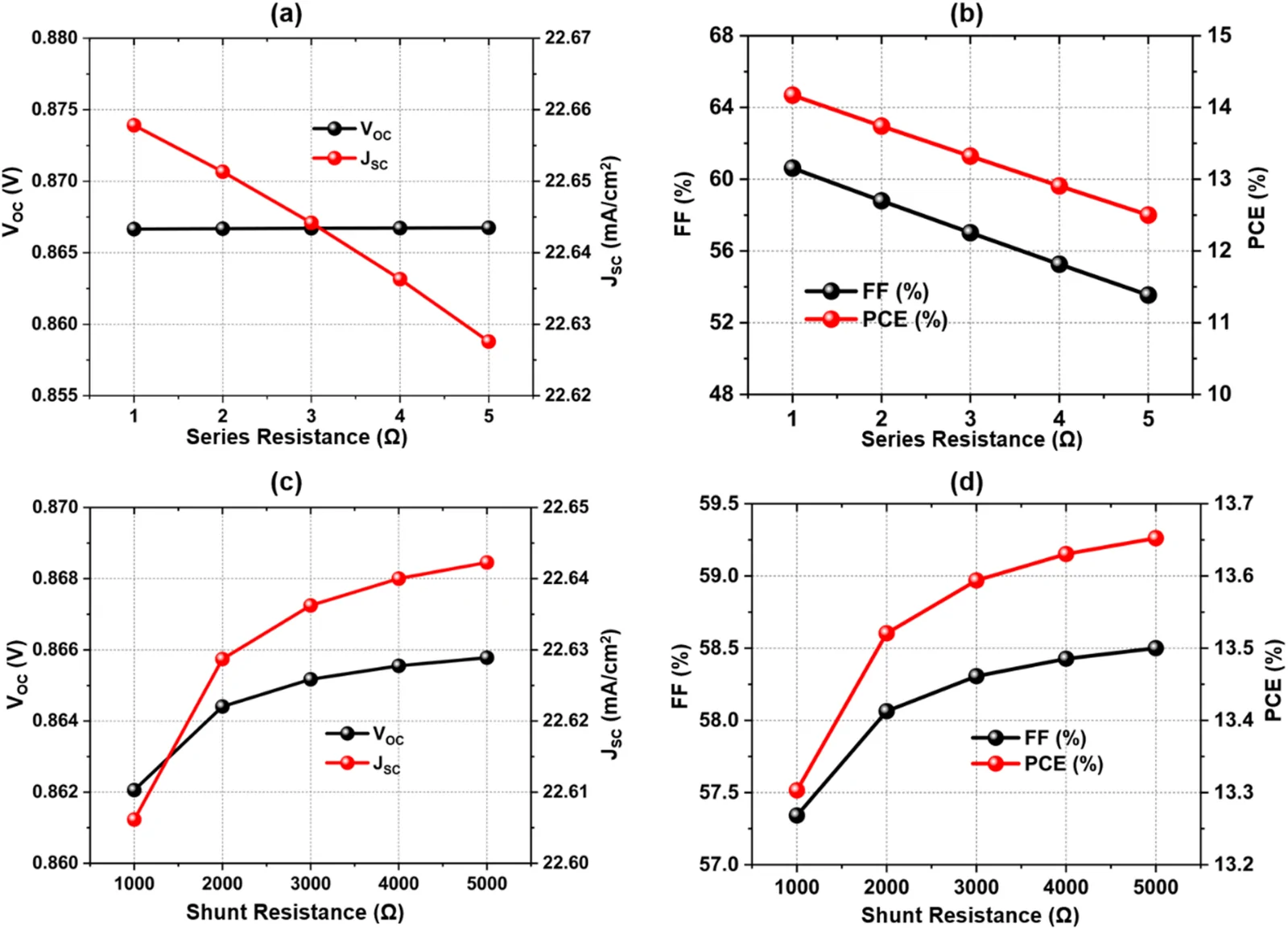
Variation of device parameter for (a and b) series and (c and d) shunt resistance.

### 2.13. Effect of temperature

Temperature strongly affects PSC performance and stability. Although PSCs generally operate near room temperature (300 K), the actual device temperature may vary depending on environmental conditions and solar irradiation. Therefore, investigating the thermal stability of the proposed Sr–ZnO/FASnI_3_-based PSC is essential for evaluating its practical applicability and long-term operational reliability. Previous studies reported that elevated temperature can increase interface defects, which ultimately deteriorate device performance.^43^ The thermal stability of the proposed device was investigated within the temperature range of 300–360 K. The obtained photovoltaic parameters are presented in Fig. 13. It can be observed that increasing temperature negatively affects the overall device performance.^73^ Among all photovoltaic parameters, *V*_OC_, FF, and PCE showed significant degradation with rising temperature, whereas *J*_SC_ exhibited only a very small variation.

**Fig. 13:**
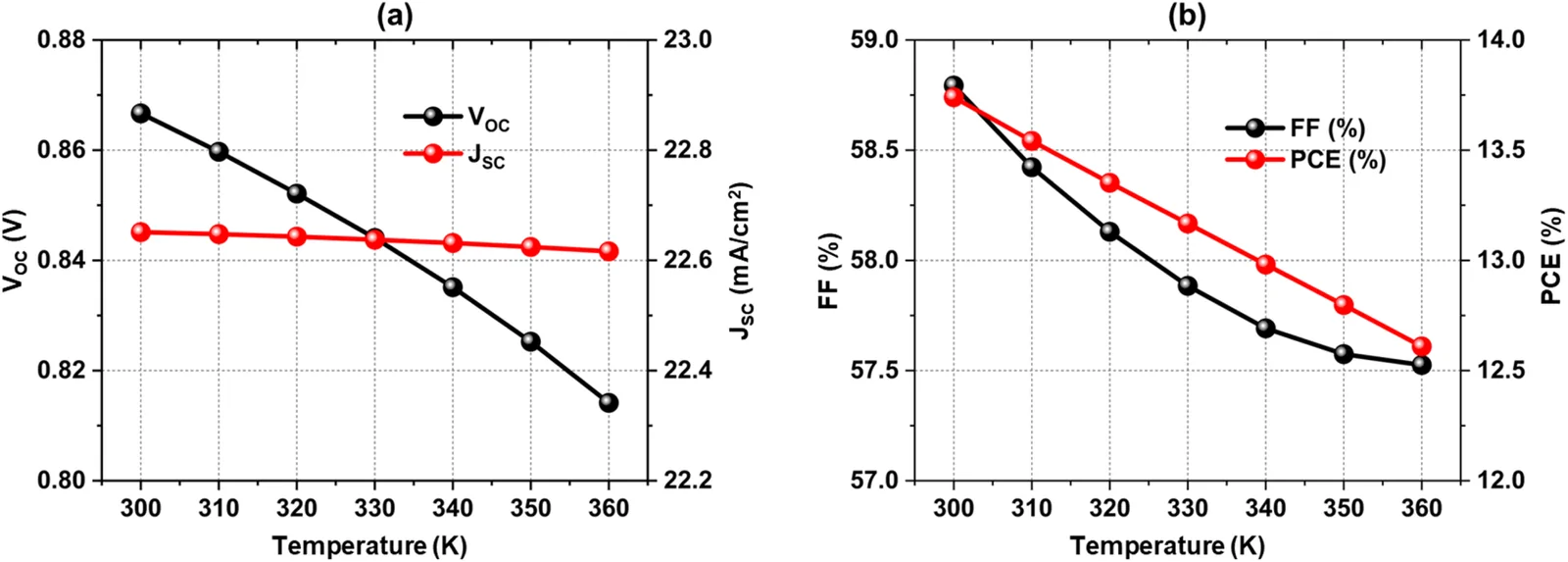
Effect of temperature on device performance. (a) *V*_OC_*vs. J*_SC_ (b) FF *vs.* PCE.

The *V*_OC_ decreased continuously from 0.8667 V at 300 K to 0.8141 V at 360 K, corresponding to an overall reduction of approximately 6.1%. This reduction occurs because higher temperature enhances carrier recombination and increases the reverse saturation current, thereby lowering the built-in potential of the device. Similarly, the FF decreased from 58.79% to 57.53%. Higher temperatures increase resistance and recombination, reducing FF. In contrast, the *J*_SC_ showed only a negligible decrease, varying from 22.65 mA cm^−2^ at 300 K to 22.62 mA cm^−2^ at 360 K. This slight variation indicates that temperature has minimal influence on the photo-generated current of the device.^89^

### 2.14. Effect of capacitance and Mott–Schottky study

Capacitance and Mott–Schottky (M–S) analyses were used to examine charge transport, depletion behaviour, and built-in potential. Fig. 14(a) shows *C*–*V* characteristics for carrier concentrations from 10^12^ to 10^20^ cm^−3^. The capacitance remains almost unchanged at low applied voltages, indicating a stable depletion region and efficient charge confinement at the junction. A sharp increase in capacitance at 0.7–0.8 V arises from increased carrier injection and interfacial charge accumulation.^90^ Furthermore, the increase in carrier concentration results in a higher capacitance response, implying improved charge storage capability and increased carrier accumulation within the depletion layer. The M–S plots shown in Fig. 14(b) were further analysed to determine the junction parameters. The linear dependence of 1/*C*^2^ on the applied voltage follows the conventional M–S relation, enabling the extraction of the built-in potential from the intercept of the fitted line on the voltage axis. The calculated *V*_bi_ (0.85–0.92 V) indicates a strong internal field for efficient charge separation, while the reduced M–S slope at higher carrier concentrations reflects increased effective doping. The slight shift in the intercept voltage further confirms that carrier concentration strongly influences the depletion width and junction barrier potential. Therefore, the *C*–*V* and M–S analyses demonstrate that optimizing carrier concentration significantly enhances junction quality, promotes efficient carrier extraction, and contributes to the improved overall photovoltaic performance of the proposed device.

**Fig. 14:**
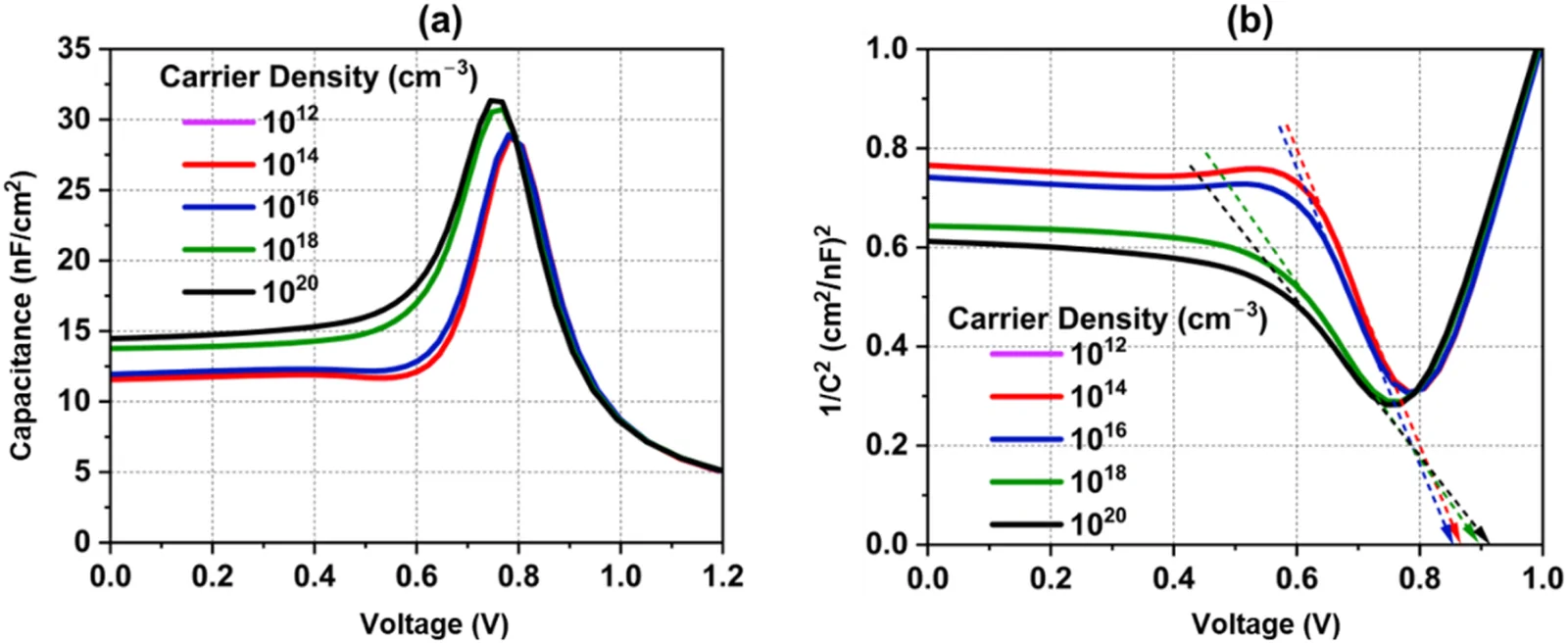
Effect of (a) capacitance and (b) Mott–Schottky analysis with different carrier density in PSC.

### 2.15. Effect of back contact electrode

The back-contact work function is a critical factor affecting PSC performance. Increasing the metal work function reduces the interfacial barrier height, resulting in improved ohmic contact formation and more efficient charge extraction. To investigate this effect, different back-contact metals with varying work functions were simulated, including Cr (4.6 eV), Ag (4.7 eV), Fe (4.8 eV), Cu (4.9 eV), C (5.0 eV), and Au (5.1 eV), as illustrated in Fig. 15. The simulation results demonstrate a gradual enhancement in device efficiency with increasing work function, where the PCE increased from 7.70% for Cr to 13.75% for Au. Specifically, the devices achieved PCE values of 7.70%, 9.76%, 11.88%, 13.48%, 13.74%, and 13.75% for work functions of 4.6, 4.7, 4.8, 4.9, 5.0, and 5.1 eV, respectively. From Section 2.8, the device exhibits the optimum photovoltaic performance, achieving 16.08%. Table 4 compares the photovoltaic performance of PSCs employing ZnO-based ETLs. Reported PCEs range from 6.52% to 23.84%, depending on the absorber, HTL, and device configuration. The proposed device structure in this study demonstrates competitive performance compared with previously reported ZnO-based PSCs. The effect of back-contact materials was investigated under the initial calibrated device conditions to understand their influence on the photovoltaic performance. Low-work-function metals can create Schottky contacts, impede carrier transport and increase recombination losses.^43^ In contrast, higher work function electrodes reduce the majority carrier barrier height and facilitate efficient hole extraction from the absorber layer. As the metal work function increases, the reduced barrier height suppresses interfacial recombination and improves charge collection efficiency. Among all investigated metals, Au exhibited the best device performance due to its high work function (5.1 eV), which enhanced both FF and PCE through favourable band alignment and efficient carrier extraction. Conversely, Cr showed the lowest efficiency because of its comparatively lower work function and the associated Schottky barrier formation. Previous studies have shown that Au can provide good photovoltaic performance as a metal electrode, while Ag and Cu-based electrodes may suffer from comparatively poor long-term stability.^91^ In particular, Ag electrodes have been reported to exhibit faster performance degradation. These findings indicate that selecting a high work function back-contact metal is essential for minimizing energy losses and achieving high-performance PSCs employing the Sr-doped ZnO ETL.

**Fig. 15:**
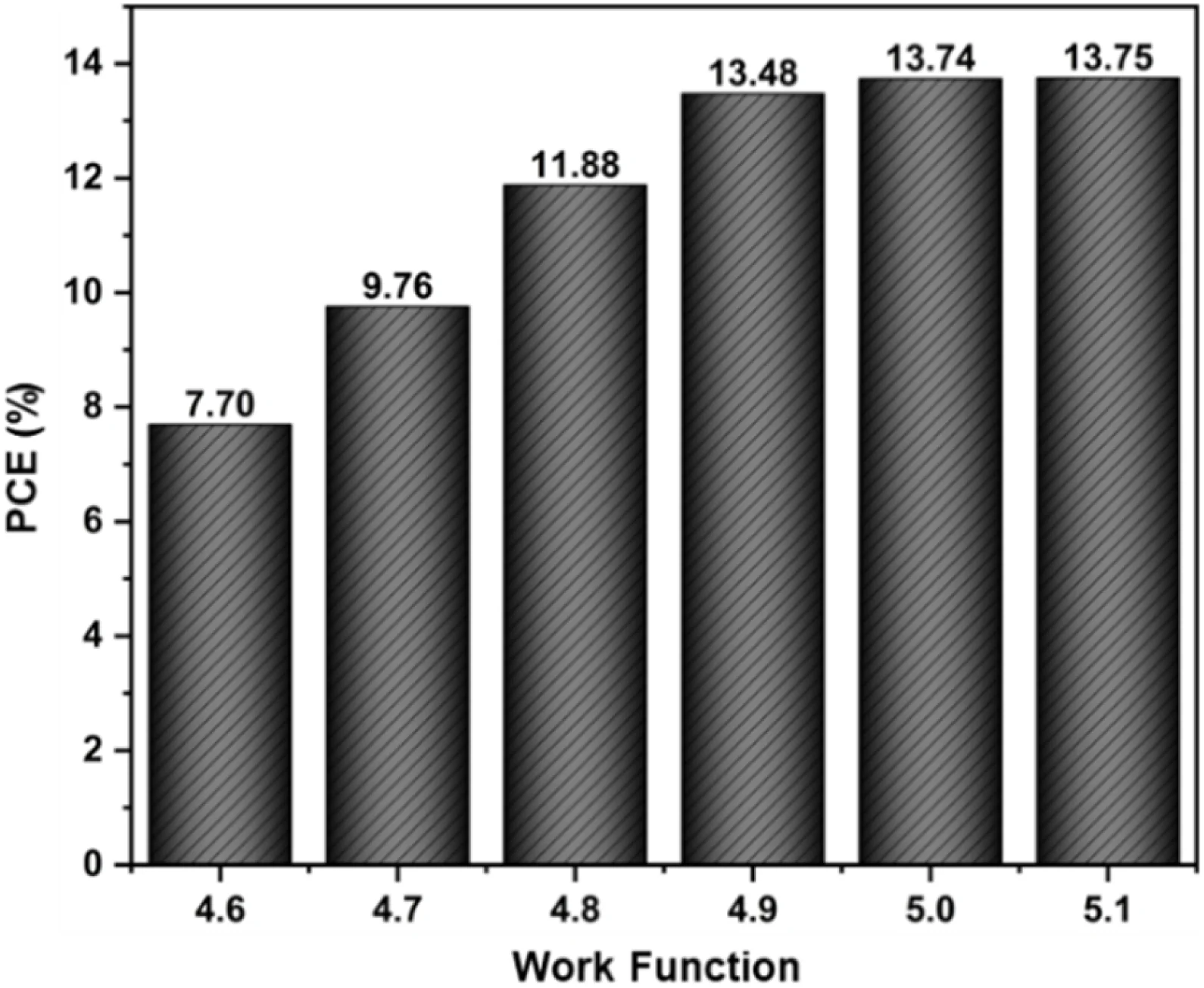
Effect of back contact electrodes on device performance.

**Table 4:** Comparison of PSC performance using ZnO ETL

Device structure	Study	*V*_OC_ (V)	*J*_SC_ (mA cm^−2^)	FF (%)	PCE (%)	Ref.
FTO/ZnO/FASnI_3_/spiro-OMeTAD/Au	Simulation	0.92	22.65	67.74	14.03	68
FTO/ZnO/CH_3_NH_3_PbI_3_/spiro-OMeTAD/Au	Experiment	0.79	27.25	0.42	10.25	92
FTO/ZnO/CH_3_NH_3_PbI_3_/PEDOT:PSS/Au	Simulation	1.27	21.78	74.53	20.64	93
FTO/ZnO/CsSnI_3_/NiO_*x*_/Au	Simulation	1.05	28.02	81.22	23.84	94
ITO/ZnO/CsPbI_3_/CBTS/Au	Simulation	0.997	21.07	85.03	17.86	95
FTO/ZnO/CH_3_NH_3_PbI_3_/CuI/Au	Simulation	0.76	32.85	71.74	17.94	96
FTO/TiO_2_/Ni–ZnO/CH_3_NH_3_PbI_3_/spiro-MeOTAD/Ag	Experiment	0.86	20.67	36.32	6.52	97
FTO/ZnO/MAPbI_3_/spiro-OMeTAD/Au	Experiment	1.04	19.31	71.09	14.25	98
FTO/ZnO/MAPbI_3_/spiro-OMeTAD/Ag	Experiment	0.97	20.4	66	13.1	99
FTO/Sr–ZnO/FASnI_3_/spiro-OMeTAD/Au	Simulation	0.89	22.64	66.86	16.08	This work

## 3 Conclusion

This work systematically investigated the suitability of Sr-doped ZnO as ETL for lead-free FASnI_3_-based PSCs through an integrated DFT and SCAPS-1D simulation approach. The DFT + *U* approach was employed to reduce the band gap underestimation inherent in conventional DFT calculations. The DFT calculations with mGGA functional demonstrated that Sr incorporation significantly modifies the electronic structure of ZnO, leading to a gradual reduction in band gap from 3.37 eV for pristine ZnO to 3.17 eV and 2.91 eV for 4.17% and 8.34% Sr doping, respectively. The DOS analysis confirmed that Sr doping enhances electronic conductivity and carrier transport. Optical analysis revealed strong UV absorption, high transparency in the visible zone, low extinction coefficient, and tunable optical properties, confirming the suitability of Sr–ZnO for photovoltaic applications. The SCAPS-1D simulation of the FTO/Sr–ZnO/FASnI_3_/spiro-OMeTAD/Au calibrated device achieved a simulated PCE of 13.74%. Optimization studies revealed that device performance strongly depends on ETL thickness, donor density, interface defect concentration, CBO, series resistance, and operating temperature. The optimum ETL configuration was obtained at a thickness of 150 nm and donor concentration of 1 × 10^20^ cm^−3^, resulting in a maximum PCE of 16.08%, with improved FF and charge transport efficiency. Furthermore, the study demonstrated that interface defects at the Sr–ZnO/FASnI_3_ junction influence carrier recombination. Increasing interface defect density from 10^12^ to 10^20^ cm^−2^ reduced the PCE from 13.74% to 8.24%, highlighting the importance of interface passivation. A small positive CBO (∼0.10 eV) offers optimal band alignment, promoting electron extraction while minimizing recombination. Thermal analysis indicated that increasing the temperature from 300 K to 360 K caused noticeable degradation in *V*_OC_, FF, and PCE, while *J*_SC_ remained nearly unchanged. Additionally, high work-function back-contact metals such as Au provided superior device performance by minimizing interfacial barrier losses. This theoretical research offers experimentalists with direction for selecting and optimizing critical material and device characteristics. These findings aid in identifying the primary performance limiting variables and providing appropriate parameter ranges prior to experimental fabrication, hence facilitating the development of higher-PCE devices based on the proposed design. Overall, results confirm Sr-doped ZnO as a promising ETL for efficient, stable, and eco-friendly lead-free PSCs. The findings provide valuable guidelines for interface engineering, defect control, and device optimization toward the future development of high-performance Pb-free photovoltaic technologies.

## Author contributions

Shamima Ahmed: writing – original draft; M. Mottakin: conceptualization, supervision & writing – review and editing; Aqib Adnan Shafin: writing and editing; Huda Abdullah: validation; Abdulaziz K. Assaifan: supervision and formal analysis.

## Conflicts of interest

There are no conflicts to declare.

## Data Availability

Data will be made available upon a reasonable request to the corresponding author.

## References

[cit1] Proskuryakova L. N., Ermolenko G. V. (2019). Renewable Energy.

[cit2] Bosio A., Pasini S., Romeo N. (2020). Coatings.

[cit3] Singh J., Agrahari A. (2019). Int. J. Energy Res..

[cit4] Andreani L. C., Bozzola A., Kowalczewski P., Liscidini M., Redorici L. (2019). Adv. Phys.:X.

[cit5] Mustafa G. M., Akhtar M. S., Sabir U., Almaymoni N. K., El-Fattah W. A., Mnif W., Iqbal M. (2026). J. Sol-Gel Sci. Technol..

[cit6] Madani S., Tesfamichael T., Wang H., Motta N. (2022). Ceram. Int..

[cit7] Kim N. H., Hyun Y. Y., Lee K. B., Chang Y., Rhu S., Oh K. H., Ahn C. (2015). J. Korean Med. Sci..

[cit8] Il Kwak J., Lee T. Y., An Y. J. (2023). J. Cleaner Prod..

[cit9] Verma A. A., Dwivedi D. K. (2025). Sci. Rep..

[cit10] Gourji F. H., Velauthapillai D. (2021). Molecules.

[cit11] Chabri I., Ouaha A., Taoussi S., Said M., Oubelkacem A., El bouari A., Bih L. (2025). Ceram. Int..

[cit12] Alkhammash H. I., Mottakin M., Hossen M. M., Akhtaruzzaman M., Rashid M. J. (2022). Semicond. Sci. Technol..

[cit13] Zhang P., Yang J., Wei S. H. (2018). J. Mater. Chem. A.

[cit14] Chen M., Ju M. G., Carl A. D., Zong Y., Grimm R. L., Gu J., Zeng X. C., Zhou Y., Padture N. P. (2018). Joule.

[cit15] Amraoui S., Feraoun A., Kerouad M. (2022). J. Alloys Compd..

[cit16] Sekar K., Manisekaran R., Nwakanma O. M., Babudurai M. (2024). Adv. Energy Sustainability Res..

[cit17] Ullah A., Khan M. I., Ihtisham-Ul-Haq N., Almutairi B. S., Dalil D. B., Choi J. R. (2024). ACS Omega.

[cit18] Farooq U., ur Rehman W., Wang Y. L., AlAahir B., Ghodhbani R. (2025). J. Sol-Gel Sci. Technol..

[cit19] Sarkar D. K., Mottakin M., Hasan A. K. M., Selvanathan V., Islam M. A., Shahiduzzaman M., Alharbi H. F., Akhtaruzzaman M. (2023). Jpn. J. Appl. Phys..

[cit20] Kotbi A., Lejeune M., Barroy P., Alaoui I. H., Zeinert A., Jouiad M. (2025). J. Sol-Gel Sci. Technol..

[cit21] Acharya A., Ye M., Kabel J., Sharma S., Asthana A., Neupane K., Uddin J., Zhang D., Yap Y. K. (2025). ACS Appl. Energy Mater..

[cit22] Ellabban O., Abu-Rub H., Blaabjerg F. (2014). Renewable Sustainable Energy Rev..

[cit23] Wang L., Giles N. C. (2003). J. Appl. Phys..

[cit24] Vispute R. D., Talyansky V., Choopun S., Sharma R. P., Venkatesan T., He M., Tang X., Halpern J. B., Spencer M. G., Li Y. X., Salamanca-Riba L. G., Iliadis A. A., Jones K. A. (1998). Appl. Phys. Lett..

[cit25] Adnan M. A. M., Julkapli N. M., Hamid S. B. A. (2016). Rev. Inorg. Chem..

[cit26] Yu W., Zhang J., Peng T. (2016). Appl. Catal., B.

[cit27] Tsay C. Y., Wu C. W., Lei C. M., Chen F. S., Lin C. K. (2010). Thin Solid Films.

[cit28] Maldonado F., Stashans A. (2010). J. Phys. Chem. Solids.

[cit29] Srinivasulu T., Saritha K., Reddy K. T. R. (2017). Mod. Electron. Mater..

[cit30] Sin J. C., Lam S. M., Lee K. T., Mohamed A. R. (2013). Ceram. Int..

[cit31] Qiu X., Li G., Sun X., Li L., Fu X. (2008). Nanotechnology.

[cit32] Water W., Yan Y. S. (2007). Thin Solid Films.

[cit33] Vijayan T. A., Chandramohan R., Valanarasu S., Thirumalai J., Subramanian S. P. (2008). J. Mater. Sci..

[cit34] Udayabhaskar R., Karthikeyan B. (2014). J. Appl. Phys..

[cit35] Ouhaibi A., Ghamnia M., Dahamni M. A., Heresanu V., Fauquet C., Tonneau D. (2018). J. Sci.:Adv. Mater. Devices.

[cit36] Raghavendra P. V., Bhat J. S., Deshpande N. G. (2017). Mater. Sci. Semicond. Process..

[cit37] Wu T., Cui D., Liu X., Meng X., Wang Y., Noda T., Segawa H., Yang X., Zhang Y., Han L. (2020). Sol. RRL.

[cit38] Yu B. B., Chen Z., Zhu Y., Wang Y., Han B., Chen G., Zhang X., Du Z., He Z. (2021). Adv. Mater..

[cit39] Cui D., Liu X., Wu T., Lin X., Luo X., Wu Y., Segawa H., Yang X., Zhang Y., Wang Y., Han L. (2021). Adv. Funct. Mater..

[cit40] Nishimura K., Kamarudin M. A., Hirotani D., Hamada K., Shen Q., Iikubo S., Minemoto T., Yoshino K., Hayase S. (2020). Nano Energy.

[cit41] Jiang X., Li H., Zhou Q., Wei Q., Wei M., Jiang L., Wang Z., Peng Z., Wang F., Zang Z., Xu K., Hou Y., Teale S., Zhou W., Si R., Gao X., Sargent E. H., Ning Z. (2021). J. Am. Chem. Soc..

[cit42] Datta S., Kaphle G. C., Baral S., Mookerjee A. (2015). J. Chem. Phys..

[cit43] Patwary M. A., Shafin A. A., Alam M. M., Durjoy R. K. S., Ludin N. A., Su’ait M. S., Akhtaruzzaman M., Mottakin M. (2025). RSC Adv..

[cit44] Burgelman M., Nollet P., Degrave S. (2000). Modelling polycrystalline semiconductor solar cells. Thin Solid Films.

[cit45] Burgelman M., Verschraegen J., Degrave S., Nollet P. (2004). Prog. Photovolt.: Res. Appl..

[cit46] Sobayel K., Rahman K. S., Karim M. R., Aijaz M. O., Dar M. A., Shar M. A., Misran H., Amin N. (2018). Chalcogenide Lett..

[cit47] Movla H. (2014). Optik.

[cit48] Javed A., Shaheen M., Shahbaz M., Ramzan M. S., Ullah R., Jiang W. (2026). Nanotechnology.

[cit49] Janotti A., Van de Walle C. G. (2011). Phys. Status Solidi B.

[cit50] Alam M. M., Durjoy R. K. S., Patwary M. A., Shafin A. A., Su’ait M. S., Shahiduzzaman M., Akhtaruzzaman M., Mottakin M. (2026). New J. Chem..

[cit51] Özgür Ü., Alivov Y. I., Liu C., Teke A., Reshchikov M. A., Doǧan S., Avrutin V., Cho S. J., Morko H. (2005). J. Appl. Phys..

[cit52] Edvinsson T. (2018). R. Soc. Open Sci..

[cit53] Kurnia F., Ng Y. H., Tang Y., Amal R., Valanoor N., Hart J. N. (2016). Cryst. Growth Des..

[cit54] Bui V. H., Nguyen Y. T. B., Doan D. T. K., Hoang H. C. (2024). VNUHCM Journal of Science and Technology Development.

[cit55] Yadav P., SanthiBhushan B., Srivastava A. (2025). Beilstein J. Nanotechnol..

[cit56] Mansouri I., Saib F., Boulahlib S., Laachachi A., Özacar M., Bessekhouad Y. (2024). Inorg. Chem. Commun..

[cit57] Agashe C., Kluth O., Hüpkes J., Zastrow U., Rech B., Wuttig M. (2004). J. Appl. Phys..

[cit58] Studenikin S. A., Golego N., Cocivera M. (2000). J. Appl. Phys..

[cit59] Cao J., Liu D., Xia W., Liu F., Chen B. (2023). IEEE J. Electron Devices Soc..

[cit60] Hong C., Kim M., Lee J. G., Shao Q., Lee H. S., Park H. H. (2023). Int. J. Energy Res..

[cit61] Mohamed A. H., Ghazali N. A. B., Chong H. M. H., Cobley R. J., Li L., Kalna K. (2020). Solid-State Electron..

[cit62] Poduval G. K., Duan L., Hossain M. A., Sang B., Zhang Y., Zou Y., Uddin A., Hoex B. (2020). Sol. RRL.

[cit63] Ikhmayies S. J. (2016). J. Electron. Mater..

[cit64] Jain N., Kumawat R., Sharma S. K. (2020). Mater. Today: Proc..

[cit65] Wu J., Ma Z., Huang S., Lei Y., Guo H., Fang Y., Xie D., Fu N., Lin Y. (2022). Chem. Eng. J..

[cit66] Wu T., Liu X., Luo X., Segawa H., Tong G., Zhang Y., Ono L. K., Qi Y., Han L. (2022). Nano-Micro Lett..

[cit67] Uddin M. S., Hossain M. K., Uddin M. B., Toki G. F. I., Ouladsmane M., Rubel M. H. K., Tishkevich D. I., Sasikumar P., Haldhar R., Pandey R. (2024). Adv. Electron. Mater..

[cit68] ul Rehman A., Afzal S., Naeem I., Bibi D., Sarwar S. G., Nabeel F., Obodo R. M. (2024). Hybrid Adv..

[cit69] ul Hassan Q., Shah S. A. A., Salman M. U., Luqman M., Mehak M., Iqbal M. J., Atiq S. (2026). Mater. Adv..

[cit70] Mottakin M., Su'Ait M. S., Chelvanathan P., Islam M. A., Shahiduzzaman M., Ibrahim M. A., Muhammad G., Akhtaruzzaman M. (2024). Semicond. Sci. Technol..

[cit71] Ahmed S., Haque M. M., Sobayel K., Alharbi H. F., Shahiduzzaman M., Ibrahim M. A., Akhtaruzzaman M. (2024). Opt. Mater..

[cit72] Maffei R. M., Butterling M., Liedke M. O., D'Addato S., di Bona A., Bertoni G., Gazzadi G. C., Mariazzi S., Wagner A., Brusa R. S., Benedetti S. (2024). Appl. Surf. Sci..

[cit73] Ouslimane T., Et-taya L., Elmaimouni L., Benami A. (2021). Heliyon.

[cit74] Mahmoud K. H., Alsubaie A. S., Anwer A. H., Ansari M. Z. (2023). Micromachines.

[cit75] Schnabel T., Seboui M., Bauer A., Choubrac L., Arzel L., Harel S., Barreau N., Ahlswede E. (2017). RSC Adv..

[cit76] Fan R., Zhou W., Huang Z., Zhou H. (2020). EnergyChem.

[cit77] Liu Q., Vandewal K. (2023). Adv. Mater..

[cit78] Wang Y., Zhu W., Chen X., Yang X., Tong A., Yang S., Wu J., Sun W. (2025). Nanoscale.

[cit79] Niu H., Fang C., Wei X., Wang H., Wan L., Li Y., Mao X., Xu J., Zhou R. (2021). Dalton Trans..

[cit80] Yang L., Xu T., Bai Z., Qin S. (2023). J. Phys. Chem. C.

[cit81] Ichihara F., Sieland F., Pang H., Philo D., Duong A. T., Chang K., Kako T., Bahnemann D. W., Ye J. (2020). J. Phys. Chem. C.

[cit82] Sekar K., Marasamy L., Mayarambakam S., Hawashin H., Nour M., Bouclé J. (2023). RSC Adv..

[cit83] Mottakin M., Sobayel K., Ismail R. C., Shahiduzzaman M., Muhammad G., Ibrahim M. A., Akhtaruzzaman M., Hossain E. S. (2022). Phys. Status Solidi RRL.

[cit84] Singh N., Agarwal A., Agarwal M. (2022). Opt. Mater..

[cit85] Lunge O. R., Ravidas B. K., Bhattarai S., Pandey R., Madan J., Roy M. K., Hossain M. K., Samajdar D. P. (2024). J. Phys. Chem. Solids.

[cit86] Mujahid A., Khan M. Y. H., Uddin M. M., Alamer F. A., Alsalmi O., Rasheduzzaman M., Hasan M. Z. (2025). RSC Adv..

[cit87] Karthick S., Velumani S., Bouclé J. (2020). Sol. Energy.

[cit88] Ali M., Khan Q., Din M. F. U., Kasi J. K., Kasi A. K., Ali A., Ullah S. (2025). Sol. Energy.

[cit89] Wang K., Gunawan O., Todorov T., Shin B., Chey S. J., Bojarczuk N. A., Mitzi D., Guha S. (2010). Appl. Phys. Lett..

[cit90] Almora O., Aranda C., Mas-Marzá E., Garcia-Belmonte G. (2016). Appl. Phys. Lett..

[cit91] Behrouznejad F., Shahbazi S., Taghavinia N., Wu H. P., Diau E. W.-G. (2016). J. Mater. Chem. A.

[cit92] Dehghan M., Behjat A. (2019). RSC Adv..

[cit93] Azri F., Meftah A., Sengouga N., Meftah A. (2019). Sol. Energy.

[cit94] Park H. J., Son H., Jeong B. S. (2024). Inorganics.

[cit95] Hossain M. K., Rubel M. H. K., Toki G. F. I., Alam I., Rahman M. F., Bencherif H. (2022). ACS Omega.

[cit96] Adeyemi O. J., Ali H., Musa I., Ushiekpan U. R., Akinade B. J., Madugu M. L. (2021). Malays. J. Appl. Sci..

[cit97] Das T., Guchhait A. (2024). Mater. Today: Proc..

[cit98] Guo Y., Kang L., Zhu M., Zhang Y., Li X., Xu P. (2018). Chem. Eng. J..

[cit99] Dong X., Hu H., Lin B., Ding J., Yuan N. (2014). Chem. Commun..

